# Value representation in youth psychopathology: evidence of a transdiagnostic risk mechanism for psychosis

**DOI:** 10.1038/s41398-026-04065-8

**Published:** 2026-06-10

**Authors:** Zachary B. Millman, James M. Gold, Jason Schiffman, Lauren M. Ellman, Elaine F. Walker, Albert R. Powers, Scott W. Woods, Steven M. Silverstein, Vijay A. Mittal, Philip R. Corlett, Gregory P. Strauss, James A. Waltz

**Affiliations:** 1 Psychotic Disorders Division, McLean Hospital, Department of Psychiatry, Harvard Medical School, Belmont, MA 02478 USA; 2 Maryland Psychiatric Research Center, Department of Psychiatry, University of Maryland School of Medicine, Baltimore, MD 21228 USA; 3 Department of Psychological Science, 4201 Social and Behavioral Sciences Gateway, University of California, Irvine, CA 92697 USA; 4 Department of Psychology & Neuroscience, Temple University, Philadelphia, PA 19122 USA; 5 Department of Psychology and Program in Neuroscience, Emory University, Atlanta, GA 30322 USA; 6 Department of Psychiatry, Yale University, New Haven, CT 06519 USA; 7 Departments of Psychiatry, Neuroscience and Ophthalmology, University of Rochester Medical Center, Rochester, NY 14642 USA; 8 Department of Psychology, Northwestern University, Evanston, IL 60208 USA; 9 Department of Psychology, University of Georgia, Athens, GA 30602 USA

**Keywords:** Diagnostic markers, Human behaviour

## Abstract

Negative symptoms of schizophrenia are associated with deficits in representing the value of actions, observed in reinforcement learning (RL) tasks as impaired learning from gains but intact learning from losses. This RL profile contrasts with depression, where enhanced loss sensitivity is common. Whether a schizophrenia-like RL pattern characterizes youth at clinical high-risk (CHR) for psychosis – who show modest psychosis transition rates but high rates of co-occurring depression – remains unclear. We tested whether CHR youth show a schizophrenia-like RL profile and whether RL parameters relate differentially to negative vs. depressive symptoms by estimating RL in CHR (*n* = 292), clinical controls (CC; *n* = 241) with other psychopathologies, and healthy controls (*n* = 175). Participants completed symptom interviews, neuropsychological assessments, a dimensional psychosis risk calculator, and an RL task in which participants could seek gains or avoid losses. A computational gain-loss *Q*-learning model decomposed task behavior into components indexing value updating and value-guided choice. Although overall RL performance was similar across groups, in both CHR and CC youth, higher negative, but not depressive symptoms, were selectively associated with reduced win-stay behavior following gains. Computational parameters suggested this behavioral pattern reflected disrupted updating and expression of positive value representations. Lower win-stay rates were also linked to lower premorbid intelligence and higher psychosis risk calculator scores. Here, a schizophrenia-like RL profile was unrelated to depression but associated with multiple indicators of psychosis risk across clinical groups, suggesting a transdiagnostic psychosis risk mechanism distinct from affective disturbance.

## Introduction

Psychotic disorders are the source of significant personal and societal burden worldwide [1]. A primary clinical contributor to disability in psychosis is negative symptoms, which interfere substantially with psychosocial functioning [2] yet respond poorly to available treatments [3]. Importantly, negative symptoms tend to emerge early in the developmental course of psychosis, even prior to the onset of positive symptoms [4], highlighting their relevance for early detection, prediction, and etiological research. The clinical high-risk (CHR) paradigm provides a valuable framework for this research by identifying adolescents and young adults with recent, brief/attenuated psychotic symptoms and/or psychosis risk factors signaling an elevated likelihood of transitioning to a formal psychotic disorder (i.e., about 25%) within 2–3 years [5]. A large proportion of youth at CHR present with negative symptoms [6], which are associated with pathogenic factors, [7, 8] functional difficulties [9], and elevated transition risk [10]. More recently, negative symptoms have been conceptualized as transdiagnostic phenomena in that they present across psychotic and non-psychotic disorders, with potentially shared and distinct etiological mechanisms and clinical implications [11].

A large body of research suggests that dysfunction of the reward system, including probabilistic reinforcement learning (RL) performance, may contribute mechanistically to negative symptoms [12, 13]. Probabilistic RL involves learning which behaviors are associated with positive outcomes when rewards are delivered at varying ratios (e.g., 70%), relying on basal ganglia-driven reward prediction error signaling in which cue-outcome associations are learned gradually and implicitly, as well as cortically-mediated, working memory-dependent mechanisms supporting explicit, prospective decision making [14]. Disturbances in RL likely contribute to difficulties initiating and sustaining goal-directed behavior, particularly when behavior must be guided by previously learned associations.

One especially informative indicator of RL is the tendency to repeat actions that were previously rewarded (“win-stay”) or to shift away from those that were not (“lose-shift”) [15]. Descriptively, these behaviors index how recent feedback influences subsequent choice, but are compatible with several underlying mechanisms, including general performance effects or altered prediction error encoding. In gain-based contexts, selectively impaired win-stay performance – particularly when accompanied by intact lose-shift performance – has been interpreted to suggest a reduced ability to generate, maintain, or use the positive expected (i.e., explicit) value of behavior [16]. Here, such value representations are understood in a computational sense to denote the latent expected value estimates inferred from behavior, rather than neural representations inferred directly [16, 17]. Computational RL models offer a principled means to constrain interpretations of task performance by parsing observed behavior into separable latent components, including parameters governing how value representations are updated following favorable or unfavorable feedback and how precisely learned values are translated into choice [17].

Multiple behavioral studies suggest that people with schizophrenia spectrum disorders (e.g., schizophrenia, schizoaffective disorder; SSDs), especially those high in negative symptoms, present with impaired win-stay but intact lose-shift rates [16, 18–25]. Computational and experimental work suggests that this pattern is not attributable to a global learning deficit per se but rather a reduced ability to use the expected value of actions in service of goal directed behavior [16, 26, 27]. Further supporting this interpretation, RL impairments are most pronounced under conditions that impose demands on cognitive control processes that support value representation. Accordingly, although SSD patients tend to perform comparably to healthy controls on RL tasks that minimize the need for explicit value representations (i.e., implicit RL tasks) [18, 28], they show reduced flexibility following reward contingency changes [20, 29] and increasing deficits as working memory demands of the environment increase [30].

The nature of reward system functioning and its relation to negative symptom presentation among youth at CHR is less clear. Several studies suggest a schizophrenia-like pattern of impairment, including reduced performance on explicit RL tasks relative to healthy controls [31–34], yet similar performance to healthy controls on more implicit RL tasks [35, 36]. It has also been reported that, as in SSDs, CHR states involve a specific impairment in learning from positive vs. negative feedback (e.g., impaired win-stay, intact lose-shift) [31, 32] and a greater tendency to learn from irrelevant stimulus features than healthy controls [33]. These behavioral similarities to SSDs are complemented by evidence of disrupted signaling in frontostriatal circuits (which subserve RL) and correlations between neurobehavioral measures of reward processing and negative symptoms in some CHR studies [31, 32, 37–41].

Despite areas of consistency in reward system function across the CHR and chronic stages of psychosis, other evidence suggests that RL impairments in CHR youth are less prominent, less selective, or even qualitatively distinct from those associated with SSDs. Some studies have reported no impairments in explicit RL in CHR youth [38, 42], potentially owing to population heterogeneity and/or small sample sizes in conjunction with true effects that may be of a smaller magnitude than is seen in established psychosis. Studies have also found that CHR and associated negative symptoms are characterized by a paradoxically *wider* range of reward system abnormalities relative to the more selective deficits seen in SSDs, including blunted hedonic reactivity [43] and simultaneous impairments in gain and loss learning [44]. These findings suggest that reward system dysfunction in CHR youth is not simply a milder version of that seen in established psychosis but may also involve additional, qualitatively distinct alterations worthy of investigation.

One hypothesis to explain the apparent divergence in reward system functioning across the CHR and chronic stages of psychosis concerns the contribution of mood symptoms. Depressive disorders co-occur in approximately 40–50% of youth at CHR [45], and reward processing impairments are well established in primary depression [46–48]. Importantly, depression is associated with a reward processing profile that differs from that observed in SSD, including relatively intact explicit RL [48–52] alongside elevated sensitivity to loss [53] and blunted hedonic reactivity [46]. Several findings in CHR samples align more closely with this depression-related configuration (altered loss learning, impaired hedonic reactivity), and reward-related impairments among CHR youth have been associated with depressive symptom severity in several studies [32, 35, 54, 55]. This mixed pattern raises the possibility that explicit RL at the CHR stage reflects the convergence of psychosis-related value representation deficits and depression-related affective mechanisms, underscoring the need to disentangle these influences when interpreting explicit RL performance in this population.

One approach to dissociating the influence of different symptom dimensions to RL is to assess the relations between RL performance and continuous measures of psychopathology while controlling for shared variance in clinical measures. This strategy provides useful information about the shared and distinct relations among measures but does not address the extent to which RL effects and its clinical correlates are observed *across* clinical populations. Transdiagnostic approaches to CHR research inclusive of clinical control (CC) groups – comprised of age-matched individuals with nonpsychotic disorders – have emerged in recent years due to their unique ability to highlight areas of commonality between clinical populations and point toward patterns more specific to psychosis risk [56–60]. The few studies adopting this stringent methodology in CHR studies of RL have in fact found similar RL performance between CHR and CC [37, 40], suggesting that associations between RL performance and symptom severity are, at least to some degree, transdiagnostic. These studies, however, generally involved small sample sizes and did not include healthy control (HC) participants, limiting reliable conclusions about deviation from normality.

The present study sought to clarify the nature of RL impairment in CHR youth and its association with key clinical and prognostic measures. In the largest CHR study of RL to date, we (1) examined patterns of WSLS behavior on an explicit RL task compared to HC as well as CC with a range of nonpsychotic presentations; (2) tested for differences in the magnitude of relations between RL and negative and depressive symptoms across clinical groups; and (3) assessed for relations between RL, premorbid intelligence, and estimated probability of transition to psychosis as determined by a validated risk calculator. Additionally, we fit a computational model to decompose task behavior into learning and value expression components to improve the mechanistic understanding of observed RL performance. We expected that although reductions in RL performance would be observed among both CHR and CC groups as well as among participants high in negative or depressive symptoms (common impairment), reduced win-stay performance following gains would be more closely related to established markers of psychosis risk (specific impairment). Moreover, we expected that reduced win-stay performance following gains would reflect disruptions in the organization and expression of learned value (rather than a global learning deficit or impaired processing of negative feedback), and that such disruptions would relate differentially to acquired value preferences between healthy vs. clinical youth.

## Materials and methods

### Study design and general procedures

This manuscript draws from the multisite Computerized Assessment of Psychosis Risk (CAPR) study [61]. The CAPR study is focused on prediction of clinical trajectories in youth using computerized, behavioral measures of psychosis risk. Participating sites include the University of Maryland, Northwestern University, University of California Irvine, Temple University, University of Georgia, University of Rochester, Emory University, and Yale University. The protocol was approved by the Northwestern University institutional review board, and all participants provided informed consent. The broader study was initiated in April 2020, with the research sessions taking place over a virtual platform, due to the COVID-19 pandemic. Participants completed clinical interviews, self-report questionnaires, and a battery of computerized behavioral tasks relevant to major symptom dimensions and mechanisms of psychosis.

### Participants

Three groups of participants ages 12–35 were recruited: individuals at CHR, clinical controls, and healthy controls. This age range is consistent with large-scale CHR studies and captures the wide range of developmental risk for psychosis [62–64]. Exclusion criteria for all participants included history of serious head injury, neurological disorder, lifetime substance or alcohol dependence, or lifetime formal psychotic disorder. Individuals at CHR met established criteria for a psychosis-risk syndrome. Healthy controls presented with no lifetime history of serious psychopathology, no current psychotropic medication use or history of antipsychotic use, and no first-degree relative with a psychotic disorder. Social anxiety disorder and mild, past substance use disorder were not exclusionary for HC in the broader protocol, but for the present analyses we excluded these individuals (*n* = 5) to ensure non-overlapping, group-specific clinical criteria across CHR, CC, and HC. Clinical controls had no CHR syndrome but presented with current and/or past non-psychotic psychopathology, including a range of disorders from the Diagnostic and Statistical Manual of Mental Disorders 5th Edition (DSM-5) and/or attenuated positive symptoms that fell short of criteria required for a CHR syndrome. Several studies have shown that these criteria capture a population with similar or greater rates of non-psychotic disorders at follow up relative to CHR yet substantially lower risk of psychosis [65–67].

### Clinical measures

Clinical high-risk status and positive symptom severity were determined using the Structured Interview for Psychosis-risk Syndromes (SIPS) [68]. Psychiatric diagnosis was determined using the Structured Clinical Interview for DSM-5 (SCID-5) [69]. Negative symptom severity was assessed using the Negative Symptom Inventory – Psychosis Risk (NSI-PR) [70], an interview-based instrument developed through extensive validation. Depression severity was measured using the 14-item version of the Center for Epidemiologic Studies Depression Scale (CES-D) [71].

Premorbid intelligence was assessed with the word reading subtest of the Wide Range Achievement Test (WRAT) [72]. The degree of two-year risk for transition to a formal psychotic disorder was assessed using the North American Prodrome Longitudinal Study (NAPLS) risk calculator [73]. The calculator incorporates clinical, historical, and neurocognitive information to produce individualized estimates of transition risk at one- and two-years following baseline assessment. In addition to having been validated on several independent CHR samples (e.g. [74]), this tool was shown to accurately predict transition to psychosis in CC with affective disorders, highlighting the potential for transdiagnostic applications [75]. Details of the clinical assessments are included in Methods S1.

### Reinforcement learning task

**Fig. 1:**
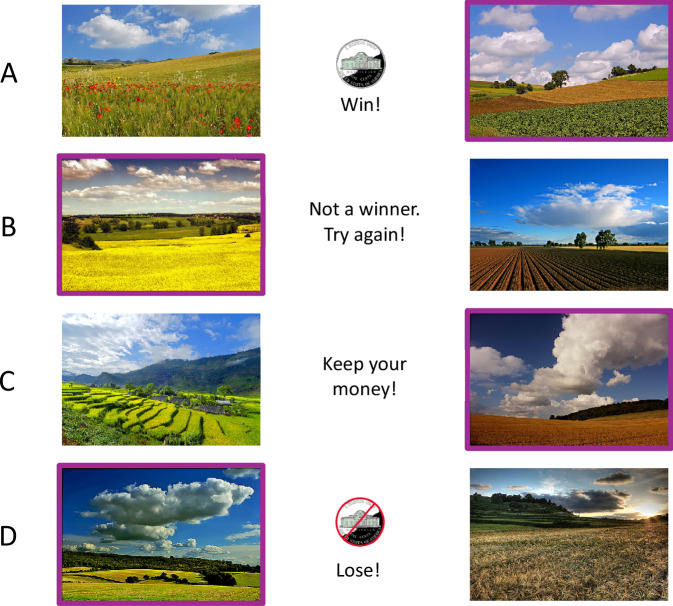
Stimuli and feedback examples of the explicit reinforcement learning task. Participants chose between two stimuli that resulted probabilistically in outcome feedback. **A** and **B** reflect trials in which a win (i.e., gain) or non-win was possible, (**C**) and (**D)** reflect trials in which an avoidance of loss or a loss was possible. Choice examples are indicated by purple border. Image adapted from Gold et al. [16].

Details regarding the remote development and administration of the behavioral tasks are provided in Methods S2. The RL task was chosen due to evidence that it is sensitive to negative symptom severity in established SSDs [16, 18, 22]. On each trial, participants were presented with one of four pairs of stimuli (landscape images; Fig. 1). Two of these pairs could lead to a gain and the other two could lead to avoidance of loss. In the gain pairs, choice of the correct stimulus was rewarded in 90% of trials in one pair (AB; Fig. 1A) and in 80% of trials in the other (CD; Fig. 1B). Correct responses resulted in an image of a nickel alongside the word “Win!”, whereas incorrect responses resulted in the words “Not a winner, Try again!”. In the loss-avoidance pairs, choice of the correct stimulus avoided the loss of a nickel in 10% of trials in one pair (EF; Fig. 1C) and 20% of trials in the other pair (GH; Fig. 1D). Correct responses would result in the words “Keep your money!”, whereas incorrect responses would result in a crossed-out nickel alongside the word “Lose!”. On each trial, the stimulus pair remained on the screening until the participant made a choice. Each response was followed by the presentation of feedback (1 s) and 1-s intertrial interval. Participants completed a practice run of 12 trials, followed by 160 learning trials with each pair presented 10 times in each of four blocks, with each pair maintaining the same reinforcement contingencies throughout the session.

After completion of this Acquisition phase, participants were presented with Transfer trials, consisting of 4 familiar and 12 novel pairings of the Acquisition stimuli. Participants were told to choose what they thought was the better stimulus and received no feedback. Familiar pairings of stimuli were the same four stimulus pairs participants had seen in the Acquisition phase. Novel pairings of stimuli included Frequent Winners vs. Frequent Losers (AF, AH, CF, CH), Frequent Winners vs. Frequent Loss-Avoiders (AE, AG, CE, CG); and Infrequent Winners vs. Frequent Loss Avoiders (BE, BG, DE, DG). Each of these 16 pairings was presented 6 times, for a total of 96 Transfer trials.

From the Acquisition phase, we calculated win-stay (percent of trials in which the participant stayed with the same choice after an optimal outcome) and lose-shift (percent of trials in which the participant shifted after a suboptimal outcome) rates separately for gain and loss-avoidance pairs, resulting in four primary behavioral variables: win-stay_G_, lose-shift_G_, win-stay_LA_, lose-shift_LA_. We also examined data from the Transfer phase. The Frequent Winner vs. Frequent Loss-Avoider contrast is of particular interest because both stimuli are associated with the same frequency of prediction errors in the Acquisition phase *but have acquired different expected values*, allowing us to assess participants’ use of expected value on choice [76–78]. Calculations of relevant parameters are described in Methods S2.

### Data analysis

#### Preprocessing and preliminary analyses

Primary study variables were assessed to determine whether they met assumptions of normality and homoscedasticity through visually inspecting histograms, means, standard deviations, and estimates of skewness and kurtosis. Demographic and clinical characteristics of the CHR, CC, and HC groups were compared using one-way analyses of variance (ANOVAs) for continuous variables (e.g., symptom severity) and chi-square tests for categorical variables (e.g., sex). See Methods S3.1 for handling and analysis of missing data and potential confounds (medication, performing for money vs. points).

#### Computational modeling

To decompose the RL mechanisms of task behavior, we fit a gain-loss *Q*-learning model to data from the task Acquisition phase as in prior work in SSDs and CHR [16, 27, 40]. Here, *Q* reflects the expected value estimate on which all model parameters operate. The model estimates separate learning rates for favorable (α_P_) and unfavorable (α_N_) outcomes, in addition to an inverse temperature parameter (β) reflecting how precisely learned values guide actions. In this formulation, α_P_ controls updating following successful gain or loss-avoidance, whereas α_N_ controls updating following omission or loss, representing the extent to which value representations are reinforced by positive feedback or are destabilized by negative feedback, respectively. Details of the model, including evaluation of its adequacy and parameter interpretability, are found in Methods S3.

#### Primary analyses

The Benjamini-Hochberg correction was used to correct for false discovery rate among hypothesis-driven analyses. Results not surviving this correction are noted. To assess for the presence of between-group difference in RL performance, we conducted one-way ANOVAs and Kruskal-Wallis tests with group (CHR, CC, HC) as the factor and WSLS parameters as dependent variables.

Within the combined clinical sample, a correlation matrix was produced to determine the relations between the primary behavioral, clinical group, and symptom variables. When evidence of a relation (*p* < 0.10) was observed between more than one RL measure and a clinical variable, we planned a sequence of increasingly stringent regression models to determine the robustness and specificity of behavior-symptom relations. Predictors in this sequence included all four WSLS variables, group (CHR/CC), and symptom severity, as well as a behavior × group term to determine differential relations of behavior to symptoms across CHR and CC. To determine the relation of RL to premorbid cognition and psychosis transition probability, we conducted correlational analyses between the RL measures surviving the steps above and scores on the WRAT and NAPLS risk calculator (Methods S4.3-S5.1).

Focal analyses of computational parameters (Methods S5.2) aimed to determine the latent mechanisms of task behavior. A linear regression model was fit with win-stay_G_ as the dependent variable and α_P_, α_N_, and β as simultaneous predictors, controlling for clinical status (HC/clinical). To determine whether win-stay_G_ is best explained by reward value updating when learned values are expressed with high precision, a regression with these same predictors plus an α_P_ × β interaction was conducted. To characterize the computational substrates of feedback-free gain vs. loss-avoidance preferences, we fit regressions with the Frequent Winner vs. Frequent Loss-Avoider contrast as the dependent variable and all three computational parameters as predictors. Separate models were fit for HC and clinical youth to provide a qualitative characterization of the data, with subsequent regression models estimating parameter × group interactions to formally assess between-group differences.

Supplementary analyses of behavioral data examined the contribution of age and sex to the relation between RL and symptoms, as well as associations between RL and separate dimensions of the NSI-PR, associations between RL and other neurocognitive measures, and learning accuracy and transfer (Methods S6). Supplementary analyses of computational data further examined inter-parameter relations as well as between-group differences in parameter values, their associations with symptoms, and the influence of age and sex on clinical parameter-symptom relations.

## Results

### Data preprocessing

The dataset included 708 individuals, including 292 at CHR, 241 CCs, and 175 HCs. Overall, listwise *n*’s among pairs of primary study variables ranged from 695 to 708 for RL variables, 499 to 527 for RL-symptom pairs (CHR and CC groups only), and 438 to 443 for RL-risk calculator pairs (CHR and CC groups only). Participants with missing data appeared largely similar to those with useable data on the demographic, clinical, and RL features, and no effects of medication exposure or task condition (money vs. points) were observed on RL (Results S1-S2, Tables S1-5). Due to extreme skewness and kurtosis, β was dichotomized at the median value (0.033).

The three groups presented with similar mean age and similar distributions of sex, ethnicity, and socioeconomic levels, with some racial differences between groups (Table 1). As expected, relative to HCs, CHR and CC youth both presented with more severe depressive and positive symptoms (negative symptoms were not assessed in HCs). Moreover, as expected the CHR group presented with greater symptom severity, higher risk calculator scores, and higher rates of medication use, relative to the CC group.

**Table 1:** Demographic Characteristics of the Sample.

Variable	CHR	CC	HC	F/X/t (df)	p
N	292	241	175	–	
Age (M (SD))	23.41 (4.2)	23.78 (4.1)	23.7 (4.15)	0.58 (2, 705)	0.561
Sex (% Female)	66	62.8	57.2	3.54 (2)	0.171
Race (%)				23.19 (6)	0.001
Asian	18.6	22.6	36.6	–	
American Indian	0.7	1.3	0	–	
Black/African American	16.1	17.1	14	–	
White	52.1	52.6	41.3	–	
Hawaiian	0.7	0	0	–	
Multiracial	11.8	6.4	8.1	–	
Hispanic (%)	14.2	14.6	9.2	3.07 (2)	0.215
Parental Education (%)				4.82 (4)	0.306
Less than high school	3.1	2.5	2.3	–	
High school or equivalent	11.7	14.9	10.4	–	
Some college	9	9.6	4	–	
Associates or 2-year college	6.2	7.1	8.1	–	
Bachelor’s degree	28.7	31.8	31.8	–	
Graduate degree	38.1	37.2	43.4	–	
Positive symptoms (SIPS)	10.87 (3.62)	3.89 (2.74)	1.87 (2.06)	604.44 (2, 701)	<0.001
Negative symptoms (NSI-PR)	15.24 (8.37)	12.61 (7.81)	–	3.634 (506)	<0.001
Depressive symptoms (CES-D)	18.76 (8.01)	14.52 (8.07)	8.06 (5.52)	106.92 (2, 671)	<0.001
Premorbid Intelligence (WRAT)	110.26 (14.55)	109.77 (14.52)	110.75 (14.6)	0.196 (2, 609)	0.822
Psychosis transition probability estimate (NAPLS risk calculator)	12.55 (8.24)	5.81 (3.03)	–	11.75 (310.21)	<0.001
Medication (% lifetime use)				–	
Antipsychotic	10.3	2.5	0	11.1 (1)	<0.001
Antidepressant	30.8	23.2	1.7	3.82 (1)	0.05
Mood stabilizer	6.5	2.5	0.6	4.77 (1)	0.03
Stimulant	8.9	6.2	0.6	1.34 (1)	0.24
Benzodiazepine	7.5	2.9	1.1	5.5 (1)	0.02
DSM-5 Diagnoses (%)					
Depressive	30.4	20.9	0	5.99 (1)	0.014
Bipolar	11.2	0.9	0	22.49 (1)	<0.001
Anxiety	65	46.6	0	17.86 (1)	<0.001
Obsessive-Compulsive	14	6.4	0	7.72 (1)	0.005
Substance	23.8	16.7	0	3.98 (1)	0.046
Trauma/Stressor	28.4	11.6	0	21.8 (1)	<0.001
ADHD	23	12.7	0	8.95 (1)	0.003

For statistical analysis, racial categories were collapsed to Asian, black/African American, white, and other ensure adequate cell counts. Parental education categories were collapsed to high school or less, some college/associates/2-year completed, and bachelor’s degree or higher. Healthy controls were excluded from chi-square analyses of lifetime medication use (due to small sample sizes), negative symptoms (as data not collected for healthy controls), and DSM-5 diagnoses (as comparisons of interest are the CHR and CC groups).

*CC* clinical control, *CHR* clinical high-risk, *HC* healthy control, *CES-D* center for epidemiological studies depression scale, *NSI-PR* negative symptom inventory – psychosis risk, *SIPS* structured interview for psychosis-risk syndromes.

### Primary analyses

**Fig. 2:**
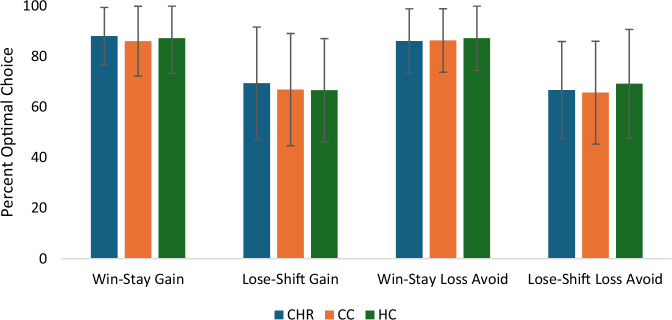
Explicit reinforcement learning performance during task learning phase across study groups. Error bars represent standard deviation. Healthy control positive error for win-stay_G_ was truncated at the maximum observed value (100) for visual depiction purposes; all numeric error values are provided in Table S6. CHR clinical high-risk, CC clinical control, HC healthy control, win-stay_G_ win-stay on gain trials.

Results indicated no difference in WSLS parameters across the groups (Fig. 2, Results S2, Table S6). The correlation matrix in the combined clinical sample (Table 2) suggested that negative symptoms were broadly associated with WSLS variables: Participants with lower win-stay_G_ or win-stay_LA_ presented with more severe negative symptoms, and small correlations trending toward statistical significance suggested a similar effect for lose-shift_G_ and lose-shift_LA_. No relation was observed between any RL measure and depressive or positive symptoms, with very small correlation coefficients (i.e., 0.004–0.051).

**Table 2:** Correlations Among Clinical and Reinforcement Learning Measures Included in Subsequent Regression Models in the Combined Clinical Sample.

	2	3	4	5	6	7	8
1. CHR status	−0.159***	−0.254***	−0.731***	−0.064	−0.057	0.004	−0.026
2. Negative symptoms	1	0.204***	0.387***	−0.139**	−0.087^a^	−0.087*	−0.075^a^
3. Depressive symptoms		1	0.279***	0.030	−0.002	0.005	0.058
4. Positive symptoms			1	0.016	0.033	−0.059	0.048
5. Win-stay_G_				1	0.383***	0.623***	0.274***
6. Lose-shift_G_					1	0.285***	0.231***
7. Win-stay_LA_						1	0.349***
8. Lose-shift_LA_							1

CHR status refers to clinical high-risk or clinical control. *Subscript G* gain trials, *subscript LA* loss-avoidance trials.

^a^*p* < 0.10, *p < 0.05, **p < 0.01, ***p < 0.001.

**Fig. 3:**
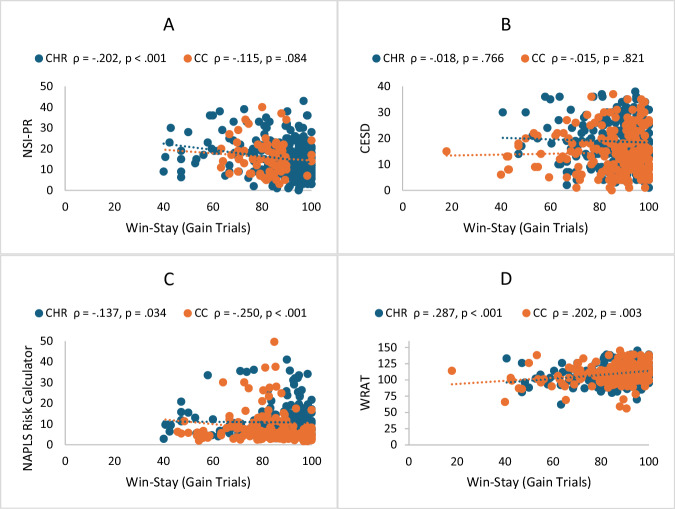
Scatter plots depicting Spearman correlations between win-stay performance on gain trials and clinical variables. **A**: Win-stay performance and negative symptom severity. **B**: Win-stay performance and depressive symptom severity. **C**: Win-stay performance and estimated probability of later psychosis transition. **D**: Win-stay performance and premorbid cognitive performance. CHR clinical high-risk, CC clinical control, CESD center for epidemiological studies depression scale (14-item), NAPLS North American Prodrome Longitudinal Study, WRAT wide range achievement test (word reading scale).

A linear regression model with negative symptom severity as the dependent variable, all four RL variables as independent variables, and group (CHR/CC) as a covariate suggested that win-stay_G_ was significantly negatively associated with negative symptom severity while controlling for the other RL variables and CHR status (Table 3). None of the other RL variables was related to negative symptoms in the model. The effect remained significant when adding depressive and positive symptom severity as covariates. A regression model including all independent variables from the prior model plus the cross-product of clinical group and win-stay_G_ indicated no group × RL interaction, suggesting the magnitude of the negative relation between win-stay_G_ and negative symptoms did not differ significantly across CHR and CC participants (CHR: ρ = −0.202, *p* = 0.001; CC: ρ = −0.115, *p* = 0.084; Fig. 3).

**Table 3:** Results of Regression Models Predicting Negative Symptom Severity from Reinforcement Learning Measures and Clinical Covariates.

Omnibus	R^2^	F (df)	p
**Model 1.**			
	0.06	5.90 (2, 487)	<0.001
Predictor	B (sb)	t	p
Win-stay_G_	−0.12 (0.04)	−2.92	0.004
Lose-shift_G_	−0.02 (0.02)	−0.82	0.441
Win-stay_LA_	0.03 (0.04)	0.85	0.398
Lose-shift_LA_	−0.02 (0.02)	−0.94	0.347
CHR status	2.89 (0.74)	3.93	<0.001
**Model 2.**			
	0.20	15.88 (7, 457)	<0.001
Predictor	B (sb)	t	p
Win-stay_G_	−0.10 (0.04)	−2.68	0.008
Lose-shift_G_	−0.02 (0.02)	−1.10	0.273
Win-stay_LA_	0.04 (0.04)	1.01	0.312
Lose-shift_LA_	−0.03 (0.02)	−1.72	0.086
CHR status	0.63 (1.03)	0.62	0.539
Positive symptoms	0.19 (0.11)	1.77	0.078
Depressive symptoms	0.35 (0.04)	8.04	<0.001
**Model 3.**			
	0.20	14.23 (8, 456)	<0.001
Predictor	B (sb)	t	p
Win-stay_G_	−0.29 (1.3)	−2.32	0.021
Lose-shift_G_	−0.02 (0.02)	−1.07	0.283
Win-stay_LA_	0.05 (0.04)	1.26	0.209
Lose-shift_LA_	−0.03 (0.02)	−1.70	0.090
CHR status	0.63 (1.03)	0.61	0.541
Positive symptoms	0.19 (0.11)	1.77	0.078
Depressive symptoms	0.35 (0.04)	7.95	<0.001
Win-stay_G_ x CHR status	0.09 (0.06)	1.58	0.115

CHR status refers to clinical high-risk or clinical control. *Subscript G* gain trials, *subscript LA* loss-avoidance trials.

Clinical participants with lower win-stay_G_ presented with lower WRAT scores (CHR: ρ = 0.287, *p* < 0.001; CC, ρ = 0.202, *p* = 0.003) and higher scores on the NAPLS risk calculator (CHR: ρ = −0.137, *p* = 0.034; CC: ρ = −0.250, *p* < 0.001; Fig. 3). The relation between win-stay_G_ and negative symptoms held when controlling for WRAT scores (*b* [0.03] = −0.10, *t* = −3.13, *p* = 0.002) and other neurocognitive measures (Results S2.3, Table S10). Supplementary analyses indicated sex and age-contingent effects of win-stay_G_ on negative symptoms, a shallower performance increase from late learning to Transfer among CHR youth vs. HC, and associations between negative symptoms and reduced preference for gain stimuli during Transfer (Results S2.4, Tables S11-12, Figure S1).

**Fig. 4:**
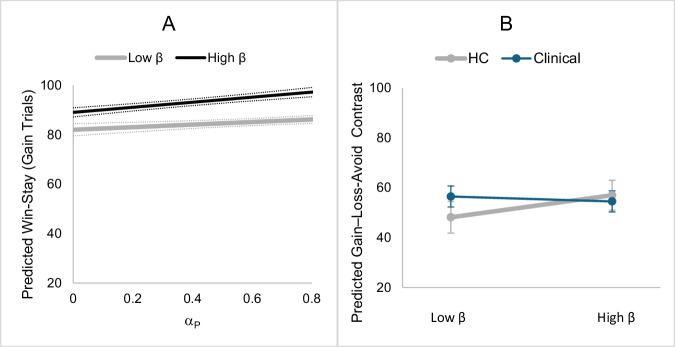
Line graphs depicting model-predicted task behavior from computational parameters. Dashed lines represent 95% confidence intervals. **A**: Interaction of α_P_ and β in prediction of win-stay performance following gains during the Acquisition phase. Predicted values plotted at the observed range. **B**: Interaction of β and clinical status in prediction of preference for Frequent Winner vs. Frequent Loss-Avoider in the Transfer phase. Values reflect the percent of feedback-free choices made favoring the stimulus associated (during the Acquisition phase) with a gain when the other option was one associated with avoidance of loss. HC healthy control.

Computational modeling further clarified the mechanisms underlying the behavioral findings. Win-stay_G_ was primarily explained by learning from favorable outcomes (α_P_) and the precision with which learned values guided choice (β), with a significant α_P_ × β interaction indicating that the α_P_–win-stay_G_ relation was substantially stronger at high vs. low levels of β (*b* [1.95] = 5.04, *t* = 2.59, *p* = 0.010); Fig. 4A). Analysis of Transfer phase data suggested that for HC, β was associated with the expected preference for Frequent Winners over Frequent Loss-Avoiders; in clinical participants, however, β was associated with a relative preference for Frequent Loss Avoiders. This pattern was confirmed by a significant β × group interaction (*b* [4.62] = −10.86, *t* = −2.35, *p* = 0.019; Fig. 4B), with similarly abnormal effects among CHR and CC (Results S4.3). Computational parameters showed sex-specific and age-contingent associations with negative symptoms, with α_N_ and β showing effects in the opposite direction (Results S5, Tables S18-20).

## Discussion

The overarching goals of this study were to characterize RL performance in CHR youth and determine the extent to which impairments in RL performance uniquely index psychosis risk or reflect broader psychopathology. To this end, we examined the relations between WSLS variables and several indicators of psychosis risk using stringent tests of specificity, accounting for the influence of broad performance effects, depressive symptoms, and general clinical status (i.e., via CC). The results suggest that across clinical groups, negative symptoms are associated with reduced learning from gains alongside intact learning from losses. Computational modeling indicated that rather than reflecting a general learning deficit, this behavior reflects a compromised ability to update internal representations of reward and to translate them into choice. Despite no detectable influence of RL on either depressive or positive symptom severity, participants with this behavioral pattern also showed lower premorbid intelligence and higher estimated probability of psychosis transition. The findings have implications for our understanding of the role of RL in negative symptom formation across clinical populations, as well as detection and prediction research in the CHR field.

A central aim of the present work was to sharpen the field’s ability to make inferences about shared vs. illness-specific effects of reward processes on CHR-related psychopathology. This was motivated by evidence that negative and depressive symptoms – both associated with reward processing in established illness – frequently co-occur in CHR youth and have been related to task behavior and functional brain activation [32, 35, 54, 55, 79]. These observations raise the question of whether reward system functioning in CHR youth is more like schizophrenia (e.g., selective impairment in gain learning) [31, 32], more like primary depression (e.g., altered loss sensitivity) [44, 54], or a mixture of both (e.g., distributed, multi-domain deficits) [43]. First, the results consistently indicated no association between depressive symptoms and any WSLS measure. Second, the effect of win-stay_G_ on negative symptoms remained robust when controlling for dimensional depression, despite strong covariance between these two symptom dimensions. Third, and critically, we found that the relation of win-stay_G_ to negative symptoms was not unique to CHR, as the same general pattern was seen in CC despite milder symptom severity. These findings argue against the hypothesis that reward system abnormalities in CHR youth are largely attributable to affective distress or hedonic dysfunction and instead suggest that selectively reduced explicit learning from gains reflects a more schizophrenia-like vulnerability; yet, this pattern extends beyond the CHR framework, suggesting a transdiagnostic risk profile for psychosis.

We found that participants with reduced win-stay_G_ had lower premorbid intelligence and higher estimated risk of psychosis transition. Importantly, these results can be considered reasonably independent of the association between RL and negative symptoms because (a) neither negative symptoms nor premorbid intelligence contribute to risk calculator scores; (b) positive symptoms (which do contribute to the risk calculator) were not associated with RL; and (c) the effect of win-stay_G_ on negative symptoms was not merely explained by lower neurocognitive ability. Although the risk calculator effects in this study were of a small magnitude and observed (vs. estimated) transition data will be essential in validating prognostic utility, these results make an important link between deficits in RL and putative clinical outcomes, highlighting the potential for accessible behavioral measures to inform prediction of trajectories [61].

Consistent with a computational account of value representation, conventionally defined as the updating or expression of value inferred from behavior [14, 16], modeling results indicated that win-stay performance depended on how positive value was updated (α_P_) and how consistently learned values were expressed in choice (β). Even when value was updated, its behavioral impact depended on the precision of expression, suggesting differences in how values are constructed and deployed rather than whether learning occurred per se. During transfer, healthy and clinical youth also showed opposing associations between choice precision and preference for previously rewarded vs. loss-avoiding stimuli: Higher β predicted greater gain preference for HC but a relative preference for loss avoidance in clinical youth. This pattern suggests that greater choice precision may amplify an existing bias away from reward in certain populations. Sensitivity analyses indicated that these effects were not specific to CHR or CC participants, consistent with a shared vulnerability. Future work comparing alternative computational models may further refine the mechanisms underlying these effects.

Supplementary analyses provide additional context for interpreting our main findings. Both win-stay_G_ and computational parameters showed sex-specific, developmentally conditional associations with negative symptoms, consistent with known sex differences in SSDs and adolescent maturation of reward systems. Although overall performance trajectories were comparable across groups, CHR youth showed a shallower increase from late Acquisition to Transfer, suggesting disruptions in learned value expression rather than general RL. Motivation and pleasure deficits were also correlated with reduced preference for reward-related stimuli during Transfer. These findings suggest that alterations in value-guided decision-making may emerge or consolidate during the transition from adolescence to young adulthood, with implications for identifying windows of vulnerability.

Despite consistent relations between RL and clinical variables, the CHR and CC groups achieved overall RL accuracy similar to that of HCs. This finding is consistent with some [38, 42] but not all [31–33] prior studies. The reasons for these mixed results are unclear but may reflect population heterogeneity, differences in sampling methodology that shape clinical characteristics, or true effects of a small magnitude. Notably, even in chronic schizophrenia, where explicit RL deficits are arguably most pronounced, the deficits have in some studies been observed only among high negative symptom individuals [16]. Nonetheless, the results suggest that the current task’s utility as a first-line screening or diagnostic measure may be limited. Alternatively, negative symptom treatments for youth could plausibly use the present task to track individual improvement in basic reward mechanisms. Replication, test-retest reliability, and sensitivity to treatment gains, however, would first need to be demonstrated in these populations.

This study had several strengths, including its large multisite sample and use of a CC group, stringent testing for effects of theoretically relevant covariates on task performance, a state-of-the-art negative symptom inventory, and a computational model to decompose behavior into latent mechanisms. The study also had some limitations. Although the remote task administration methodology was carefully developed and tested, participant performance was monitored in real time, and none of the performance variables were based on reaction time, most studies of RL in this population have administered tasks in person, which may affect generalizability of results or comparison with prior literature. Moreover, negative and depressive symptoms were assessed using different methods (interview and questionnaire, respectively); although the CESD is a widely validated measure of depression severity and had good reliability in the present sample, the contribution of method variance to the findings cannot be ruled out. Our protocol also lacked a dedicated working memory test, which would have strengthened inferences about SSD-like mechanisms. We also note that the prognostic validity of NAPLS risk calculator, though previously shown to generalize to non-CHR youth with depressive disorders [75], has not yet been tested in additional CC populations. Thus, the implications for future psychosis conversion among CC should be considered cautiously until further calculator validation is performed.

In summary, this study found that a schizophrenia-like RL profile was associated with several markers of psychosis risk, including negative symptoms, premorbid intelligence estimates, and scores on a psychosis transition risk calculator. This behavioral pattern reflected a combination of reduced organization and expression of learned values and was observed across CHR youth and lower-risk CC. The findings provide evidence that explicit RL may relate to psychosis-related phenomena in the high-risk stages of illness but also among youth with less clinically discernable risk for the disorder. Important future directions include longitudinal follow-up to determine the predictive power of baseline RL performance on trajectories; studies recruiting CC with more specific syndromes (e.g., active mood episodes) to inform understanding of where reward system alterations overlap and diverge across emerging psychopathology; integration of reward tasks that show more reliable associations with depression (e.g., implicit RL) than negative symptoms; and combining data from tasks spanning relevant neurobehavioral systems to predict clinical trajectories in high-risk youth.

## Supplementary information

Supplemental Material

## Data Availability

Deidentified data can be found at the National Institute of Mental Health National Data Archive (nda.nih.gov, grant reference R01MH120088). Code for the computational model can be found at https://github.com/jawaltz/Pessig_Model.
