# Supplementary material for: Value representation in youth psychopathology: evidence of a transdiagnostic risk mechanism for psychosis
Source: Transl Psychiatry. 2026 Jun 10;16:413. doi: 10.1038/s41398-026-04065-8 (PMC13473504; doi:10.1038/s41398-026-04065-8)
Supplement: Supplementary file 1 [file 41398_2026_4065_MOESM1_ESM.docx]

**Supplementary Information for**

**Value representation in youth psychopathology:**

**Evidence of a transdiagnostic risk mechanism for psychosis**

Zachary B. Millman^1^, James M. Gold^2^, Jason Schiffman^3^, Lauren M. Ellman^4^, Elaine F. Walker^5^,

Albert R. Powers^6^, Scott W. Woods^6^, Steven M. Silverstein^7^, Vijay A. Mittal^8^, Philip R. Corlett^6^, Gregory P. Strauss^9^, James A. Waltz^2^

^1^ Psychotic Disorders Division, McLean Hospital, Department of Psychiatry, Harvard Medical School, Belmont, MA, 02478, USA

^2^ Maryland Psychiatric Research Center, Department of Psychiatry, University of Maryland School of Medicine, Baltimore, MD, 21228, USA

^3^ Department of Psychological Science, 4201 Social and Behavioral Sciences Gateway, University of California, Irvine, CA, 92697, USA

^4^ Department of Psychology & Neuroscience, Temple University, Philadelphia, PA, 19122, USA

^5^ Department of Psychology and Program in Neuroscience, Emory University, Atlanta, GA, 30322, USA

^6^ Department of Psychiatry, Yale University, New Haven, CT, 06519, USA

^7^ Departments of Psychiatry, Neuroscience and Ophthalmology, University of Rochester Medical Center, Rochester, NY, 14642, USA

^8^ Department of Psychology, Northwestern University, Evanston, IL, 60208, USA

^9^ Departments of Psychology and Neuroscience, University of Georgia, Athens, GA, 30602, USA

**Supplementary Methods**

1. **Clinical Instruments**

***1.1. Positive Symptoms.*** The Structured Interview for Psychosis-risk syndromes (SIPS) was used to rate positive symptom severity. The SIPS includes five attenuated positive symptom items rated on a 0-6 (absent to severe) scale, with scores of 3-5 indicating risk symptoms, scores of 6 indicating psychotic intensity, and scores of 1-2 indicating mild symptoms below the risk threshold. The sum of these five items was used as an indicator of past-month positive symptom severity. Because attenuated psychosis-like experiences present transdiagnostically – including among youth with non-CHR psychopathology – it is expected that CC present with scores on this scale that are higher than HC but lower than CHR^1^.

All clinical interviews were administered by advanced doctoral students or postdoctoral researchers. Interviewers obtained formal SIPS certification from the instrument’s authors and were internally trained to reliability on both the SIPS and the SCID (kappa > .80). Cases were presented at weekly multisite conferences led by a SIPS expert (JS) to establish consensus on CHR status and severity ratings.

***1.2. Negative Symptoms.*** The Negative Symptom Inventory – Psychosis Risk (NSI-PR), informed by the National Institute of Mental Health Consensus Meeting on Negative Symptoms, was developed to address conceptual and psychometric limitations of other negative symptom measures in high-risk youth populations. It includes 11 items shown through structural analyses to load onto two super-ordinate dimensions (motivation and pleasure, diminished expression) and five lower-level factors (anhedonia, avolition, asociality, alogia, and blunted affect)^2,3^. Symptoms are rated from the past week and were summed to create a measure of negative symptom severity. For supplementary analyses, we calculated scores for the two super-ordinate dimensions, motivation and pleasure and diminished expression. Notably, the NSI-PR has improved discriminant validity regarding depressive symptoms relative to the SIPS^3^. Healthy controls were not administered the NSI-PR. Training included a 2-hour introductory video conducted by the scale creators, as well as rating 3 practice videos. Raters were required to score weighted kappa > .80 in relation to gold standard rating videos.

***1.3. Depressive Symptoms.*** Items for the 14-item Center for Epidemiological Studies Depression Scale (CES-D) load onto anhedonia, affect, and somatic symptomatology. Items were summed to create a measure of depressive symptom severity. Cronbach’s alpha in the present sample was α = .89.

***1.4. Premorbid Cognition.*** The word reading subtest of the Wide Range Achievement Test (WRAT) was used to estimate premorbid intelligence. Participants are presented with a list of words ranging in difficulty and are asked to read the words aloud. Errors are documented and scores (number of correct responses) are standardized to a population mean of 100 and standard deviation of 15.

***1.5. Psychosis Transition Risk Calculator***. The degree of two-year risk for transition to a formal psychotic disorder was assessed using the risk calculator developed by the North American Prodrome Longitudinal Study (NAPLS), which is freely available online (<https://riskcalc.org/napls/>). The study introducing the risk calculator reported that it strongly discriminates between CHR youth who do vs. do not transition to psychosis over two years (C-index of .71)^4^. Moreover, an international validation study found that the risk calculator maintained strong predictive validity when applied in diverse clinical research settings and in samples with multiple risk enrichment levels, including among non-CHR youth with depressive syndromes^5^. These findings indicate that individual differences in the NAPLS psychosis risk signature can reflect psychosis vulnerability even in non-CHR clinical populations.

The risk calculator includes the following measures: age (years); SIPS positive symptom items of unusual thought content and suspiciousness (with scores 0-2 coded 0 and scores 3-6 scored 1-4, respectively); processing speed measured with the Symbol Coding test from the Brief Assessment of Cognition for Schizophrenia (raw score)^6^; verbal memory measured with the Hopkins Verbal Learning Test Revised (raw sum of trials 1-3)^7^, stressful life events measured with the Psychiatric Epidemiology Research Interview Life Events Scale (sum of 31 negative events from the past year)^8^; family history of psychosis in first-degree relatives determined through clinical interview (present/absent); past-year change in social functioning measured with the Global Functioning Social Scale (difference between current and past-year highest scores)^9^, and trauma exposure measured with the Childhood Trauma and Abuse Scale (number of trauma types endorsed)^10^. All measures required for the risk calculator were administered in the CAPR study, and the publicly available equation used to produce individual risk estimates was reproduced to calculate individual participant scores.

2. **Reinforcement Learning (RL) Task**

Due to the ongoing global COVID-19 pandemic, an online platform was developed to administer CAPR behavioral tasks remotely. The tasks were piloted prior to initiating recruitment to ensure feasibility of administration and validity of task data. A research assistant provided task instructions and monitored participant performance using screen sharing for the entirety of task administration. Participants were required to keep their camera on for the duration of the session and to maintain screen sharing at all times while the computerized task was running. The research assistant kept their video camera on while reading task instructions and answering participant questions; during task performance, however, the research assistant’s camera and microphone were turned off to minimize distractions. Throughout task administration, participant performance was closely monitored for any evidence of distraction or failure to follow task instructions. If the participant appeared distracted during task performance, the research assistant intervened by instructing the participant to eliminate the distraction (e.g., if the participant’s cellular device was making noise, they were instructed to mute the device). Research assistants could use judgment to reschedule the behavioral session in the rare event that the distraction persisted.

None of the variables of interest are based on reaction time such that performance would depend on variability of the participant’s device. To determine the potential for the task to be translated to applied, non-laboratory settings (i.e., as a potential risk assessment tool), half of the participants were administered the standard version with monetary reward feedback and the other half (counterbalanced) a version in which feedback was represented in points.

For the Transfer/Test phase of the task, we calculated the proportion of choices made for the optimal stimulus among (a) familiar pairings (AB, CD, EF, and GH in Figure 1 of the main text) and (b) novel pairings (AE, AG, CE, and CG). For these, variables reflected preference for stimuli that, during the learning phase: frequently led to gains when the other stimulus in the pair frequently led to loss (Frequent Winner vs. Frequent Loser); frequently led to gains when the other stimulus in the pair frequently led to avoidance of loss (Frequent Winner vs. Frequent Loss Avoider); and for stimuli that frequently led to loss avoidance when the other stimulus in the pair infrequently led to gain (Frequent Loss Avoider vs. Infrequent Winner).

3. **Computational Model**

We fit a gain-loss *Q*-learning model consistent with our prior work in SSDs and CHR^11,12^. Here, *Q* represents learned expectations about the value of actions and serves as the substrate for choice. All model parameters construct, modify, or express *Q*, more directly indexing mechanisms of value representation than behavioral parameters alone.

Model parameters were adjusted to maximize the probability of participants’ choices in the model. The model calculated choice likelihood according to action values for each trial. $Q_{i}\left( t \right)$ was initialized to 0 and $r\left( t \right)$ was set to +1 for positive feedback in the case of the Gain/Miss stimulus pair (AB) and 0 for positive feedback and -1 for negative feedback in the case of the Loss/Loss-avoid pair (CD). After each trial, the value of the chosen action was updated as a function of the prediction error. The gain-loss *Q*-learning model is implemented by using separate learning rates α_P_ and α_N_ for positive and negative prediction errors (α_P_ and α_N_, respectively). For each trial *t*, the expected value *Q* for the chosen stimulus *i* is updated as a function of learning rate ($\alpha_{P}$ or $\alpha_{N}$) and actual outcome $r(t)$ such that

(1) if $r\left( t \right)> 0$, $Q_{i}\left( t+1 \right)= Q_{i}\left( t \right)+ \alpha_{P}\cdot\left[ r\left( t \right)-Q_{i}\left( t \right) \right]$

(2) if $r(t) = 0$, $Q_{i}(t+1) = Q_{i}(t) + \alpha_{N}\cdot[r(t)-Q_{i}(t)]$.

According to the softmax function, the probability of choosing stimulus $i$ over the other stimuli in trial $t$ is defined as:

(3)$P_{i} (t) = \frac{exp[\beta\cdot Q_{i}(t)]}{\sum_{k=1}^{n} exp[\beta\cdot Q_{k}(t)]}$

where $n$ is the total number of choices per trial and $\beta$ is the inverse temperature reflecting response stochasticity (i.e., randomness).

We fit the model to each individual’s Acquisition data and a group of best-fit parameters (α_P_, α_N_, and β) was identified by optimizing log-likelihood estimate (LLE), where LLE is defined as the log product of all probabilities,

$LLE =log[\prod_{t} P_{i}(t)]$.

We evaluated the model comparison using Akaike’s Information Criterion (AIC), which estimates the complexity of a given model used to represent the data:

$$AIC = -2\cdot LLE + 2\cdot k$$

where *k* denotes the number of free parameters and lower AIC values indicate superior model fit. To benchmark model performance against chance behavior, an AIC value was also computed for a null model assuming random responding (AIC_0_) based on LLE_0_. When a candidate model yielded AIC > AIC_0_, the improvement in fit was insufficient to offset the added model complexity, indicating that the observed behavior was no better accounted for than random responding. Participants whose data met the criterion AIC < AIC_0_ were therefore classified as exhibiting better-than-chance behavior, whereas those failing to meet this criterion were classified as exhibiting “at-chance” behavior.

As an additional measure of fit, we calculated values for pseudo-R^2^, defined as

(LLE − r)/r,

where *r* is the log likelihood of the observed data under a model reflecting purely random choices (i.e., *p* = .5 for all choices; Daw et al, 2006). Pseudo-R^2^ statistics are unrelated to the number of trials to be fit in each set and indicate how well the model fits the data compared to one predicting chance performance.

The model parameters of interest were α_P_, α_N_, and β. Computationally, these parameters reflect rate of learning from positive (α_P_) and negative (α_N_) feedback, and choice stochasticity (β), with higher values of α reflect greater learning, and higher values of β reflect greater systematicity. Here, we emphasize their functional consequences for value representation: Higher α_P_ strengthens and stabilizes values following positive outcomes; higher α_N_ destabilizes or erodes values following negative outcomes; and β indexes the precision and consistency with which values are translated into choice. Of note, these parameters reflect computational strategy rather than capacity or intrinsic (mal)adaptation. Their contribution to adaptive behavior depends on factors such as environmental volatility, developmental stage, and how strategy components interact with one another in context (e.g., higher β is adaptive when values are accurately learned, but may reflect premature rigidity when values are poorly learned or unstable).

4. **General Data Analytic Procedures**

***4.1.* *Missing Data and Potential Confounds.*** We compared RL performance across participant subgroups with missing vs. non-missing data on key clinical variables (e.g., NSI-PR) by inspecting subgroup means, standard deviations, and frequencies of WSLS parameters, and with inferential statistics when subgroups had ≥ 20 participants. The influence on task performance of missingness and potential confounds, including psychiatric medication use and task condition (performing for money vs. points), was examined with *t*-tests. When all subgroups contained *n* ≥ 20 participants, we also performed ANOVAs to assess for missingness × group and confound × group interactions.

For symptom severity measures, missing values were imputed as the participant-level mean of available items on the same scale if > 50% of its items were complete. Participants with ≤ 50% of items completed on a given scale were excluded. Risk calculator scores were calculated only for participants with complete data on all eight components. Trial-wise task data were available for all participants; however, win-stay variables cannot be calculated for participants who never made the optimal choice and lose-shift variables cannot be calculated for participants who never made the sub-optimal choice; such participants were excluded. Analyses were performed pairwise to include all participants with available data for a given analysis.

***4.2.* *Computational* *Model Adequacy and Parameter Diagnostics.*** Tests of computational model adequacy at the level of individual participants were required to interpret individual differences in computational parameters in subsequent analyses. Pseudo-R^2^ (described further below) was used for this purpose. Participants with a pseudo-R^2^ ≥ .05 were included in analyses involving model parameters. We compared participants with values above and below this cutoff on demographic (age, sex) and clinical (group status, symptom severity, neurocognitive performance) variables to determine the extent to which the selected participants were representative of the full sample.

**4.3. *Conventions Across Major Analyses.*** The major analyses in this study involved (1) analyses of variance to test for between-group differences in variables of interest and block-wise performance across the task; (2) Pearson (for normally distributed continuous variables), Spearman (for variables with skewness or kurtosis estimates > |2|), and point-biserial correlations (for dichotomous and continuous variables) to test for within-group relations; and (3) multivariate linear regression analyses to test for dimensional relations between key measures while controlling for a range of covariates. When interaction terms were included in regression models, model terms were centered and significant effects were followed up with simple slopes analyses following Aiken and West^13^. Because win-stay_G_ constituted our primary behavioral parameter of interest and was the sole WSLS variable surviving stringent multivariate regressions, subsequent analyses of behavioral data focused on win-stay_G_ (rather than other WSLS measures).

If assumptions regarding homogeneity of variance were violated (e.g., via Levene’s test), test statistics and/or degrees of freedom were appropriately adjusted. All tests of statistical significance were two-sided. Statistical power analysis suggested that with *N* = 708 and a significance level of α = .05, observed power was 85.3%–91.2% to detect small effects (*d* = 0.25) and exceeded 99% to detect medium effects (*d* = 0.50).

5. **Detailed Description of Primary Analyses**

***5.1. Primary Behavioral Analyses.*** Regarding the initial correlation matrix estimating the interrelations of primary behavioral, clinical group, and symptom variables, the primary results of interest at this step were the correlations between RL measures and negative and depressive symptoms; relations among RL variables, among clinical variables, and between RL and positive symptoms were considered as covariates in following steps.

Hypotheses regarding the influence of behavioral RL measures on symptom severity were tested with a series of increasingly stringent linear regressions. The base model included dimensional symptom severity (negative or depressive) as the dependent variable, all four WSLS parameters as simultaneous independent variables, and group (CHR/CC) as a covariate. These models estimate the independent effects of each behavioral parameter on symptom severity while controlling for CHR status. Then, to control for covariance between symptom measures, we fit linear regression models to estimate the effects of RL on symptom severity (e.g., negative symptoms) while controlling for the other to symptom dimensions (i.e., depressive and positive symptoms) and clinical group. Following these steps, we fit a regression model, as in the prior step, but also including an RL × group term as a predictor. To provide an intuitive description of within group (CHR, CC) RL-symptom relations, we calculated correlation coefficients for these variables when effects were observed in regression models or were related to the primary study aims (e.g., RL-depression relation separately by group).

***5.2. Primary Computational Modeling Analyses.*** The primary aim of the computational modeling analyses was to decompose observed task behavior into latent computational substrates. Because these analyses have direct relevance for all study groups, they were performed on the pooled study sample, with dichotomized group status (coded 0, HC or 1, clinical) included as a covariate.

For the Acquisition phase, the primary regression models (described in the Methods of the main text) focused on decomposing win-stay_G_, as this parameter was of greatest interest to our aims. If win-stay_G_ indexes the ability to calculate, store, and/or deploy information about reward value, it should be computationally explainable by the updating of positive value representations rather than the erosion of negative ones. Moreover, this effect should be expressed most stably when the consistency and precision with which learned values are translated to choice is high. Therefore, we expected a stronger effect of α_P_ than of α_N_ on win-stay_G_, and that the association between α_P_ and win-stay_G_ would be moderated by β. Relations of computational parameters to other WSLS variables were assessed in supplementary analyses (Supplementary Methods section 6.2 below).

A key test of a transdiagnostic value representation deficit in youth psychopathology is whether CHR and/or CC youth show an altered influence of computational parameters on their preference for previously reward vs. loss-avoiding stimuli. As described in the main text, to test this hypothesis we first fit separate linear regression models for HC and the combined clinical sample, with the Transfer-phase Frequent Winner vs. Frequent Loss-Avoider variable as the dependent variable and α_P_, α_N_, and β as simultaneous predictors. This stratified approach provided a qualitative characterization of how learning and value expression parameters relate to preference formation within each group, without imposing equality constraints across groups. Observed between-group differences in the pattern of associations were then formally evaluated by including group-by-parameter interaction terms, providing a statistically conservative test of whether computational predictors of Transfer behavior differed across HC and clinical youth.

6. **Planned Supplementary Analyses**

Supplementary analyses sought to determine (1) behavioral robustness of primary analyses, including tests of global task accuracy, specificity, and demographic (i.e., age, sex) influences on RL-symptom relations; (2) interrelations among all computational parameters and between these parameters and WSLS measures; and (3) clinical and demographic relevance of the computational parameters. Unless otherwise specified, regression models predicting symptom severity scores from RL variables included CHR status, depressive (or negative) symptoms, and positive symptoms as covariates. Demographic covariates, when included, were age and sex.

***6.1.* *Behavioral Analyses*.** Supplementary behavioral analyses examined the confounding and/or moderating effects of age and sex on the relation between win-stay_G_ and negative symptoms, controlling for behavioral and clinical covariates. Correlation analyses explored whether behavioral parameters correlated differentially with the two super-ordinate NSI-PR dimensions (motivation and pleasure symptoms, expressive symptoms). Correlation and regression analyses assessed whether the relation between RL and negative symptoms is accounted for by shared covariance with neurocognitive measures. No direct measure of working memory was available.

We examined learning accuracy and generalization across groups based on RL behavioral data using a repeated measures ANOVA with valence (gain vs. loss avoidance), probability (90% vs. 80%), and block (1-4) as within-subjects factors, and study group (CHR, CC, HC) as a between-subjects factor. We then conducted a repeated-measures ANOVA with the same factors as above but including accuracy (i.e., choice of optimal stimulus) on familiar pairs, during the subsequent transfer phase, as a fifth level of the “block” factor.

Finally, we compared the three clinical groups on their stimulus choices among novel pairings in the transfer phase alone and examined correlations between these transfer phase choice variables and negative symptoms.

***6.2.* *Computational Modeling Analyses*.** Correlation analyses described the zero-order associations among computational parameters and between these parameters and all four WSLS measures. As with our focused analysis of the computational substrates of win-stay_G_, we conducted separate regression analyses with each of the remaining WSLS parameters as dependent variables and all three computational parameters as predictors, controlling for group status (HC vs. clinical). Clinically oriented analyses tested for between-group differences in model parameter values and within-group relations between model parameters and negative and depressive symptomatology. As in supplementary analyses of win-stay_G_, we tested for the confounding and/or moderating effects of age and sex on the relation between model parameters and negative symptom severity.

**Supplementary Results**

1. **Missing Data and Potential Confounds**

The number of participants with incomplete data for the symptom measures of interest were as follows: NSI-PR, *n* = 48 (20 CHR, 28 CC); CES-D, *n* = 34 (16 CHR, 12 CC, 6 HC); SIPS positive symptoms, *n* = 8 (3 CHR, 5 CC). Following imputation, *n* = 25 participants were missing NSI-PR data, *n* = 4 were missing SIPS positive symptom data, and *n* = 34 were missing CESD data (all participants with missing CES-D data were missing all 14 items). No participants were missing WRAT data and *n* = 90 clinical participants (53 CHR, 37 CC) were missing risk calculator data. The number of participants with incomplete RL data was as follows: lose-shift_G_, *n* = 9 (4 CHR, 2 CC, 3 HC); win-stay_LA_, *n* = 5 (1 CHR, 1 CC, 3 HC); lose-shift_LA_, *n* = 13 (5 CHR, 4 CC, 4 HC). Table S1 shows the results of analyses assessing demographic differences across participants with missing vs. useable data; no significant between-group differences were observed.

Tables S2-S5 show the results of analyses seeking to determine the potential influence of data missingness, medication history (antipsychotic, antidepressant, mood stabilizer, stimulant, benzodiazepine), and task condition (playing for money vs. points per randomized protocol) on RL performance. As noted above, analyses were conducted when all cells had at least 20 participants. The results suggest none of the analyzed nuisance factors had a significant influence on RL.

2. **Behavioral Robustness, Specificity, and Developmental Effects*.***

Table S6 shows the numeric means, standard deviations, and results of statistical tests assessing differences in WSLS parameters across the three groups. No significant differences were observed.

***2.1. Sex and Developmental Moderation.*** The effect of win-stay-_G_ on negative symptom severity remained significant when adding age and sex as additional covariates to the regression model. When entering age and sex as moderators of the relation between win-stay-_G_ and negative symptoms, however, a significant three-way interaction was observed. Simple effects analyses revealed that age modified the effect of win-stay_G_ on symptom severity for males but not females. Among females, the effect of win-stay_G_ was negative and fairly consistent across ages. For males, however, win-stay_G_ showed a U-shaped relation with negative symptoms across ages, reflected by a non-significant positive effect on negative symptoms at younger ages, no effect at the mean age, and a statistically significant negative effect at older ages. The results are presented in Table S7. No moderating effect of age and sex was observed for depression severity (Table S8).

***2.2. Negative Symptom Factors.*** Win-stay_G_ was correlated with both the Motivation and Pleasure and the Diminished Expression dimensions of the NSI-PR. Lose-shift_LA_ and, at trend level, Lose-shift_G_, were correlated with the Motivation and Pleasure factor. No other statistically significant correlations were observed (Table S9).

***2.3. Neurocognitive Variables.*** Win-stay_G_ was statistically significantly correlated with BACS (ρ = .188, *p* < .001) and HVLT scores (ρ = .271, *p* < .001), such that participants with higher win-stay rates on gain trials presented with higher neurocognitive performance scores. Table S10 shows the results of regression analyses indicating that win-stay_G_ remained significantly related to negative symptom severity when accounting for neurocognitive performance.

***2.4. Trial-by-Trial Performance and Transfer Phase.*** The results of the repeated measures ANOVA for Acquisition phase performance (Blocks 1-4) showed the expected main effects of block (better performance over time), probability (better performance in 90% vs. 80% conditions), and valence (better performance in gain vs. loss conditions), but no significant effect of group or interactions between group and block, probability, or valence (or higher-order interactions). That is, learning trajectories during the Acquisition phase were similar across study groups. A trend toward a statistically significant group × valence interaction was observed, with visual inspection of means and graphical plots suggesting that compared to HC and CC, the CHR exhibited a somewhat steeper performance decline in loss-avoidance relative to gain conditions. However, this was due to a slightly (and non-significantly) higher performance in the gain conditions among CHR versus the other groups.

Including transfer data in the model as the fifth level of the block factor revealed the same pattern of main effects (and trending group × valence interaction of the same nature) but with a significant group × block interaction. Post hoc comparisons suggested that this interaction was driven by a shallower performance increase from block 4 of the Acquisition/Training phase to the Test/Transfer phase among CHR youth (mean difference = 2.60, *p* < .001) relative to HC (mean difference = 5.04, *p* < .001). No other significant interactions between study group and task parameters were observed. The full results of both models are presented in Table S11 and Figure S1**.**

As shown in Table S12**,** no between-group differences were observed in the frequent winner vs. frequent loser, frequent winner vs. frequent loss avoider, or infrequent winner vs. frequent loss avoider contrasts generated from the transfer phase. However, small but statistically significant negative correlations indicated that clinical participants with more severe negative symptoms showed a reduced preference for frequent winner over frequent loser stimuli, as well as reduced preference for infrequent winner vs. frequent loss avoider stimuli. These findings were driven by the motivation and pleasure factor rather than the diminished expression factor of the NSI-PR.

3. **Model Adequacy and Parameter Diagnostics**

The quality control analyses in this section sought to determine the extent to which the computational model was adequate and yielded interpretable parameters. Of the 708 participants, pseudo-R^2^ was ≥ .05 (indicating adequate model fit) for *n* = 656 (92.7%), and the remaining *n* = 52 (7.3%) had pseudo-R^2^ < .05 (indicating poor model fit for a small minority). Model fit status was unrelated to study group (𝜒2[2] = 1.03, *p* = .597), age (t[706] = 0.76, p = .449), sex (𝜒2[2] = 0.01, *p* = .908), or the severity of positive (t[702] = -0.10, *p* = .928) or depressive symptoms (t[7] = 0.76, *p* = .449). Participants with poor model fit had higher mean negative symptom (t[506] = 3.92, *p* < .001) and lower premorbid intelligence scores (t[610] = -3.21, *p* = .001) than participants with adequate model fit, which is consistent with prior studies^14^ and is unsurprising given that negative symptoms and cognitive ability can shape task engagement and therefore the fits of structured decision-making models. Further exploration of this pattern indicated that although pseudo-R^2^ was dimensionally related to negative symptom severity across all clinical participants (*r* = -.123. *p* = .006), no relation was observed among only the participants with adequate model fits (*r* = -.055, *p* = .234). Pseudo-R^2^ was correlated with premorbid intelligence in the full sample (*r* = .240, *p* < .001) and among only those with adequate model fits (*r* = .216, *p* < .001).

Collectively, these results suggest that pseudo-R^2^ primarily reflects the level of task engagement and structure required for reliable estimation of RL parameters rather than a proxy for clinical severity. The current approach prioritizes interpretability of participant-level computational parameters while retaining a clinically representative sample, despite with results most generalizable to participants whose task performance is sufficiently structured to be captured by the RL model. The major study findings regarding the WSLS variables were replicated in the sample of participants used in subsequent analyses of computational parameters, suggesting the main findings of behavioral data were stable.

Normality estimates indicated that α_P_ and α_N_ were approximately normally distributed, whereas β showed evidence of extreme non-normality (skewness = 4.10, kurtosis = 15.67). This distribution motivated a categorical treatment to reduce statistical leverage of extreme values; thus, β was recoded based on a median split at .0331 (*n* = 328 participants classified as low β, *n* = 328 participants classified as high β). The continuous and dichotomized β variables were highly correlated (ρ = .866, *p* < .001).

4. **Computational Structure and Behavioral Grounding**

The analyses in this section sought to determine the interrelations of computational parameters and their patterns of relation to observed task behavior.

***4.1. Parameter Interrelations and Influences on Win-Stay/Lose-Shift Behavior.*** Correlational analyses indicated that α_P_ and α_N_ were positively related to one another, whereas β was negatively related to both α parameters (Table S13). This is consistent with the known tradeoff between value precision and prediction error signaling^15,16^, as outcomes are more surprising (higher α) when choice systematicity is low (lower β). Computational parameters were broadly associated with WSLS measures in correlation analyses (Table S13).

The Results of the main text and Table S14-15 report the findings linear regressions predicting win-stay_G_ from α_P_, α_N_ and β, controlling for clinical status (HC, clinical), and from these same terms plus the α_P_ × β interaction. The zero-order correlation between α_P_ and win-stay_G_ was ρ = .259 (*p* < .001) for low β and ρ = .628 (*p* < .001) for high β*.* These correlation analyses provide an intuitive description of the results of the α_P_ × β interaction described in the Results of the main text. Repeating multivariate regression analysis for the remaining WSLS variables suggested a generally predominant contribution of α_P_ and β in gain trials and a stronger, positive predictive role for α_N_ on loss-avoidance rather than gain trials. For gain trials, α_N_ was more weakly and negatively associated with behavior (Table S14)**.**

***4.3. Computational Influences on Transfer Behavior.*** Within-group regression models predicting Transfer phase preference for Frequent Winner vs. Frequent Loss-Avoider stimuli from the three computational parameters showed that α_P_ was significantly associated with preference in clinical youth but showed no evidence of an effect in HC, whereas α_N_ demonstrated a trend-level negative association in clinical youth and no association in HC. In contrast, β was the only parameter to exhibit clear, qualitatively opposing associations across groups, indicating a divergence in the expression of learned value. Thus, subsequent confirmatory tests focused on a single, theory driven moderated regression examining whether the association between β and Frequent Winner vs. Frequent Loss-Avoider stimuli preference differed by group. Controlling for α_P_ and α_N_, the regression model revealed a significant group × β effect, confirming that β exerted a differential effect on gain–loss-avoidance preference in HC vs. clinical participants (Table S16 and Figure 4 of the main text). Sensitivity analyses suggested the interaction effect was present for both CHR (*b* = -11.39 [5.08], *t* = -2.24, *p* = .025) and CC (*b* = -10.25 [5.31], *t* = -1.93, *p* = .054), with no differences between the two clinical groups (*b* = 1.01[4.63], *t* = 0.22, *p* = .827).

5. **Clinical and Developmental Relevance of Computational Parameters.**

The analyses in this section sought to determine the extent to which computational parameters relate to group status and symptomatology, and whether parameter-symptom relations depend on sex or developmental stage.

Overall, the CHR, CC, and HC groups presented with similar values of α_P_, α_N_, and β (Table S17). Computational parameters showed no zero-order relations to negative or depressive symptoms in the clinical sample (Table S17), and a linear regression model with symptom severity as the dependent variable all three model terms as independent variables plus clinical and demographic covariates indicated a similar pattern (Tables S18-S20).

In moderated regression analyses, the computational parameters showed sex-specific, developmentally conditional associations with negative symptom severity, characterized by age-dependent sign reversals that differed between males and females (Tables S18-S20).

For α_N_, a statistically significant α_N_ × age × sex interaction was observed. Simple effects analyses indicated that for males, lower α_N_ was associated with greater symptom severity at younger ages, whereas higher α_N_ was associated with greater severity at older ages. In contrast, females showed the opposite developmental pattern, with higher α_N_ relating to greater symptom severity at younger ages and lower α_N_ relating to greater severity at older ages, although these within-female effects did not reach statistical significance despite the significant three-way interaction.

Models including β as the independent variable revealed a parallel age- and sex-dependent pattern, but with effects in the opposite direction to those seen for α_N_. Among males, higher β was associated with greater negative symptom severity at younger ages and lower severity at older ages. Females again showed a qualitatively reversed but weaker age-dependent pattern that did not reach statistical significance.

For α_P_, the omnibus effect was significant, but the α_P_ × age × sex interaction did not reach conventional statistical significance (*p* = .0553). Exploratory analyses suggested a modest negative relation between α_P_ and negative symptom severity for younger males, which attenuated further as age increased. No age-dependent effects were observed among females.

No age- and sex-modified effects of model parameters were observed in the prediction of depression severity.

**Supplementary References**

1	Millman, Z. B. *et al.* Perceived social stress and symptom severity among help-seeking adolescents with versus without clinical high-risk for psychosis. *Schizophr Res* **192**, 364-370 (2018). <https://doi.org/10.1016/j.schres.2017.06.002>

2	Strauss, G. P., Walker, E. F., Carter, N. T., Luther, L. & Mittal, V. A. The Negative Symptom Inventory-Psychosis Risk (NSI-PR): Psychometric Validation of the Final 11-Item Version. *Schizophr Bull* (2024). <https://doi.org/10.1093/schbul/sbae206>

3	Strauss, G. P. *et al.* Development and Validation of the Negative Symptom Inventory-Psychosis Risk. *Schizophr Bull* **49**, 1205-1216 (2023). <https://doi.org/10.1093/schbul/sbad038>

4	Cannon, T. D. *et al.* An Individualized Risk Calculator for Research in Prodromal Psychosis. *Am J Psychiatry* **173**, 980-988 (2016). <https://doi.org/10.1176/appi.ajp.2016.15070890>

5	Koutsouleris, N. *et al.* Toward Generalizable and Transdiagnostic Tools for Psychosis Prediction: An Independent Validation and Improvement of the NAPLS-2 Risk Calculator in the Multisite PRONIA Cohort. *Biol Psychiatry* **90**, 632-642 (2021). <https://doi.org/10.1016/j.biopsych.2021.06.023>

6	Keefe, R. S. *et al.* The Brief Assessment of Cognition in Schizophrenia: reliability, sensitivity, and comparison with a standard neurocognitive battery. *Schizophr Res* **68**, 283-297 (2004). <https://doi.org/10.1016/j.schres.2003.09.011>

7	Benedict, R. H., Schretlen, D., Groninger, L. & Brandt, J. Hopkins Verbal Learning Test–Revised: Normative data and analysis of inter-form and test-retest reliability. *The Clinical Neuropsychologist* **12**, 43-55 (1998).

8	Dohrenwend, B. S., Krasnoff, L., Askenasy, A. R. & Dohrenwend, B. P. Exemplification of a method for scaling life events: the Peri Life Events Scale. *J Health Soc Behav* **19**, 205-229 (1978).

9	Cornblatt, B. A. *et al.* Preliminary findings for two new measures of social and role functioning in the prodromal phase of schizophrenia. *Schizophr Bull* **33**, 688-702 (2007). <https://doi.org/10.1093/schbul/sbm029>

10	Janssen, I. *et al.* Childhood abuse as a risk factor for psychotic experiences. *Acta Psychiatr Scand* **109**, 38-45 (2004). <https://doi.org/10.1046/j.0001-690x.2003.00217.x>

11	Waltz, J. A. *et al.* Reinforcement learning performance and risk for psychosis in youth. *The Journal of Nervous and Mental Disease* **203**, 919-926 (2015).

12	Waltz, J. A. *et al.* Motivational Deficits in Schizophrenia Are Associated With Reduced Differentiation Between Gain and Loss-Avoidance Feedback in the Striatum. *Biol Psychiatry Cogn Neurosci Neuroimaging* **3**, 239-247 (2018). <https://doi.org/10.1016/j.bpsc.2017.07.008>

13	Aiken, L. S., West, S. G. & Reno, R. R. *Multiple regression: Testing and interpreting interactions*. (Sage, 1991).

14	Dowd, E. C., Frank, M. J., Collins, A., Gold, J. M. & Barch, D. M. Probabilistic Reinforcement Learning in Patients With Schizophrenia: Relationships to Anhedonia and Avolition. *Biol Psychiatry Cogn Neurosci Neuroimaging* **1**, 460-473 (2016). <https://doi.org/10.1016/j.bpsc.2016.05.005>

15	Daw, N. D., Gershman, S. J., Seymour, B., Dayan, P. & Dolan, R. J. Model-based influences on humans' choices and striatal prediction errors. *Neuron* **69**, 1204-1215 (2011). <https://doi.org/10.1016/j.neuron.2011.02.027>

16	Daw, N. D., Niv, Y. & Dayan, P. Uncertainty-based competition between prefrontal and dorsolateral striatal systems for behavioral control. *Nat Neurosci* **8**, 1704-1711 (2005). <https://doi.org/10.1038/nn1560>

**Abbreviations for Supplementary Tables**

**Group Status**

CHR, clinical high-risk

CC, clinical control

HC, healthy control

**Symptom Measures**

NSI-PR, Negative Symptom Inventory – Psychosis Risk

CESD, Center for Epidemiologic Studies Depression scale

**Neurocognitive Measures**

BACS, Behavioral Assessment of Cognition for Schizophrenia, Symbol Coding Test

HVLT, Hopkins Verbal Learning Test

WRAT, Wide Range Achievement Test

**Acquisition Phase Parameters**

Win-stay_G_/Lose-shift_G_, behavior on gain trials

Win-stay_LA_/Lose-shift_LA_, behavior on loss-avoidance trials

α_P_, updating following positive feedback

α_N_, updating following negative feedback

**Transfer Phase Parameters**

FW, Frequent winner

FL, Frequent loser

IW, Infrequent winner

FLA, Frequent loss-avoider

**Table S1. Descriptive Statistics and Results of Between-Group Analyses for Demographic Measures Across Participants with Missing vs. Non-Missing Data on Key Variables.**

| **Variable** |  |  |  |  |
| --- | --- | --- | --- | --- |
|  | **Mean (SD) or N (%)** | | **Test Statistic (df)** | **p** |
| **NSI-PR** | | | | |
|  | **Missing**  **(n = 25)** | **Not Missing**  **(n = 508)** |  |  |
| **Age** | 23.36 (4.53) | 23.59 (4.1) | 0.27 (531) | .790 |
| **Sex (% F)** | 62.5 | 64.6 | 0.72 (3) | .869 |
| **Race (% W)** | 47.8 | 52.5 | 0.20 (1) | .658 |
| **Ethnicity (% H)** | 20.8 | 14.1 | - | - |
| **Parental education (% ≥ bachelor’s)** | 75 | 67.5 | 0.60 (1) | .440 |
| **CESD** | | | | |
|  | **Missing**  **(n = 28)** | **Not Missing**  **(n = 505)** |  |  |
| **Age** | 24.74 (5.06) | 23.51 (4.05) | -1.54 (28.96) | .216 |
| **Sex (% F)** | 56 | 64.9 | 0.83 (1) | .362 |
| **Race (% W)** | 52.4 | 52.3 | 0.0 (1) | .997 |
| **Ethnicity (% H)** | 20 | 14.1 | - | - |
| **Parental education (% ≥ bachelor’s)** | 60 | 68 | 0.73 (1) | .392 |
| **NAPLS Risk Calculator** | | | | |
|  | **Missing**  **(n = 90)** | **Not Missing**  **(n = 443)** |  |  |
| **Age** | 23.96 (4.18) | 23.5 (4.11) | -0.96 (531) | .340 |
| **Sex (% F)** | 58 | 65 | 1.98 (1) | .159 |
| **Race (% W)** | 51.8 | 46.9 | 0.69 (1) | .408 |
| **Ethnicity (% H)** | 11.4 | 15 | 0.8 (1) | .371 |
| **Parental education (% ≥ bachelor’s)** | 73 | 66.7 | 1.34 | 2.47 |

*Note*. Due to small cell sizes, race was collapsed into white and “other,” and parental education was collapsed into bachelor’s degree or higher vs. less than bachelor’s degree. Insufficient cell sizes remained for analysis of ethnicity data under NSI-PR and CESD missingness. Age displayed unequal variances in the CESD analysis, thus corrected degrees of freedom are reported.

**Table S2. Descriptive Statistics and Results of Between-Group and Interaction Analyses for Behavioral Parameters Across Participants with Missing vs. Non-Missing Data on Key Variables.**

| **Parameter** | **Mean (SD)** | | **Test Statistic (df)** | **p** | **Test Statistic**  **(df)** | **p** |
| --- | --- | --- | --- | --- | --- | --- |
|  |  |  | **Missingness** | | **Group × Missingness** | |
| **NSI-PR** | | | | | | |
|  | **Missing**  **(n = 25)** | **Not Missing**  **(n = 508)** |  |  |  |  |
| **Win-stay_G_** | 87.71 (12.87) | 87.15 (12.56) | 0.18 (N/A) | .855 | - | - |
| **Lose-shift_G_** | 66.82 (21.02) | 68.36 (21.76) | 0.34 (525) | .731 | - | - |
| **Win-stay_LA_** | 86.17 (12.65) | 86.17 (12.65) | -0.54 (529) | .589 | - | - |
| **Lose-shift_LA_** | 63.97 (17.50) | 66.38 (19.85) | 0.62 (522) | .536 | 0.25 (3, 520) | .619 |
| **CESD** | | | | | | |
|  | **Missing**  **(n = 28)** | **Not Missing**  **(n = 505)** |  |  |  |  |
| **Win-stay_G_** | 89.17 (11.48) | 87.09 (12.62) | 0.89 (N/A) | .375 | 0.06 (3, 529) | .812 |
| **Lose-shift_G_** | 72.85 (26.17) | 68.03 (21.40) | -1.14 (525) | .253 | 0.56 (3, 523) | .454 |
| **Win-stay_LA_** | 88.76 (8.98) | 86.10 (12.83) | -1.09 (529) | .279 | 0.40 (3, 527) | .525 |
| **Lose-shift_LA_** | 63.97 (17.50) | 66.38 (19.85) | 0.62 (522) | .536 | 0.25 (3, 520) | .619 |
| **NAPLS Risk Calculator** | | | | | | |
|  | **Missing**  **(n = 90)** | **Not Missing**  **(n = 443)** |  |  |  |  |
| **Win-stay_G_** | 87.13 (11.63) | 87.21 (12.76) | -0.40 (N/A) | .689 | 1.31 (3, 529) | .253 |
| **Lose-shift_G_** | 68.90 (11.63) | 68.16 (21.35) | -0.29 (525) | .773 | 0.36 (3, 523) | .551 |
| **Win-stay_LA_** | 87.56 (12.86) | 85.97 (12.62) | -1.08 (529) | .283 | 1.03 (3, 527) | .310 |
| **Lose-shift_LA_** | 67.31 (21.0) | 66.05 (19.49) | -0.54 (522) | .589 | 1.0 (3, 520) | .317 |

*Note*. Analyses were performed when all cells met or exceeded *n* = 20 participants. Analyses exclude healthy controls as the clinical variables of interest concern the clinical high-risk and clinical control groups. Table rows in which degrees of freedom are marked as “N/A” reflect Mann-Whitney U tests (z-transformed). No participants were missing data on the Wide Range Achievement Test, thus no missingness analyses were conducted.

**Table S3. Descriptive Statistics and Results of Between-Group Analyses for Behavioral Parameters Across Medication Status.**

| **Variable** | **Lifetime Medication** | **No Lifetime Medication** |  |  |
| --- | --- | --- | --- | --- |
|  | **Mean (SD)** | | **T/z (df)** | **p** |
| **Antipsychotic** |  |  |  |  |
| Win-stay_G_ | 87.91 (10.92) | 87.14 (12.68) | 0.20 (N/A) | .843 |
| Lose-shift_G_ | 66.61 (19.74) | 68.41 (21.86) | 0.48 (531) | .720 |
| Win-stay_LA_ | 87.62 (10.64) | 86.14 (12.8) | -0.69 (529) | .492 |
| Lose-shift_LA_ | 68.84 (20.25) | 66.06 (19.7) | -0.83 (522) | .410 |
| **Antidepressant** |  |  |  |  |
| Win-stay_G_ | 87.16 (12.69) | 87.21 (12.53) | 0.01 (N/A) | .992 |
| Lose-shift_G_ | 66.04 (20.36) | 69.13 (22.16) | 1.45 (525) | .146 |
| Win-stay_LA_ | 87.56 (12.34) | 85.75 (12.76) | -1.47 (529) | .143 |
| Lose-shift_LA_ | 67.85 (20.98) | 65.66 (19.23) | -1.13 (522) | .259 |
| **Mood stabilizer** |  |  |  |  |
| Win-stay_G_ | 87.49 (13.99) | 87.18 (12.50) | 0.50 (N/A) | .616 |
| Lose-shift_G_ | 62.5 (23.44) | 68.56 (21.6) | 1.34 (525) | .182 |
| Win-stay_LA_ | 86.03 (13.91) | 86.25 (12.61) | 0.08 (529) | .935 |
| Lose-shift_LA_ | 68.54 (19.19) | 66.15 (19.77) | -0.59 (522) | .554 |
| **Stimulant** |  |  |  |  |
| Win-stay_G_ | 86.21 (12.31) | 87.28 (12.59) | -0.88 (N/A) | .382 |
| Lose-shift_G_ | 66.25 (22.25) | 68.46 (21.67) | 0.63 (525) | .531 |
| Win-stay_LA_ | 83.8 (14.2) | 86.44 (12.52) | 1.29 (529) | .199 |
| Lose-shift_LA_ | 70.81 (21.42) | 65.88 (19.56) | -1.52 (522) | .130 |
| **Benzodiazepine** |  |  |  |  |
| Win-stay_G_ | 90.04 (8.35) | 87.03 (12.75) | 0.92 (N/A) | .356 |
| Lose-shift_G_ | 64.58 (28.29) | 68.49 (21.29) | 0.93 (525) | .354 |
| Win-stay_LA_ | 87.72 (11) | 86.15 (12.76) | -0.69 (529) | .490 |
| Lose-shift_LA_ | 67.57 (21.82) | 66.18 (19.62) | -0.37 (522) | .714 |

*Note.* Comparison of groups on win-stay_G_ involved Mann-Whitney U tests (*z* of U statistic reported).

**Table S4. Results of Analyses Assessing Interactions Between Clinical Group and Antidepressant Medication History on Behavioral Parameters**

| **Parameter** | **F (df)** | **p** |
| --- | --- | --- |
| **Win-stay_G_** | .044 (1, 529) | .508 |
| **Lose-shift_G_** | 0.83 (1, 523) | .364 |
| **Win-stay_LA_** | 0.14 (1, 527) | .710 |
| **Lose-shift_LA_** | 0.80 (1, 520) | .371 |

*Note*. Too few participants were missing data regarding antipsychotic, mood stabilizer, and benzodiazepine use to conduct interaction analyses, thus only analyses on antidepressant use were conducted.

**Table S5. Descriptive Statistics and Results of Between-Group and Interaction Analyses for Behavioral Parameters Across Participants Playing for Points or Money.**

| **Variable** | **Played for Points** | **Played for Money** |  |  |
| --- | --- | --- | --- | --- |
|  | **Mean (SD)** | | **Test Statistic (df)** | **p** |
| **Win-stay_G_** |  |  |  |  |
| Condition | 87.36 (12.25) | 87.06 (13.54) | 0.24 | .811 |
| Group × Condition | - | - | 1.36 (5, 702) | .257 |
| **Lose-shift_G_** |  |  |  |  |
| Condition | 68.54 (20.70) | 67.21 (22.11) | 0.83 (697) | .409 |
| Group × Condition | - | - | 1.28 (5, 693) | .278 |
| **Win-stay_LA_** |  |  |  |  |
| Condition | 86.92 (12.25) | 86.05 (13.12) | 0.91 (701) | .362 |
| Group × Condition | - | - | 2.97 (5, 697) | .052 |
| **Lose-shift_LA_** |  |  |  |  |
| Condition | 66.58 (19.80) | 67.43 (29.63) | -0.55 (693) | .583 |
| Group × Condition | - | - | 0.33 (5, 689) | .719 |

*Note.* Test statistics reflect *t* values for condition (points vs. money) analyses, and *F* values for group **×** condition analyses.

**Table S6. Descriptive Statistics and Results of Between-Group Analyses for Behavioral Parameters Across Clinical Groups.**

| **Parameter** | **CHR** | **CC** | **HC** |  |  |
| --- | --- | --- | --- | --- | --- |
|  | **Mean (SD)** | | | **Test Statistic (df)** | **p** |
| **Win-stay_G_** | 88.11 (11.34) | 86.09 (13.84) | 87.26 (13.88) | 3.169 (2, 705) | .205 |
| **Lose-shift_G_** | 69.41 (22.25) | 66.93 (22.21) | 66.68 (20.44) | 1.246 (2, 696) | .288 |
| **Win-stay_LA_** | 86.13 (12.76) | 86.37 (12.57) | 87.27 (12.75) | 0.453 (2, 700) | .636 |
| **Lose-shift_LA_** | 66.72 (19.22) | 65.71 (20.36) | 69.26 (21.47) | 1.586 (2, 692) | .205 |

*Note*. Between-group differences in win-stay_G_ were determined using the Kruskal-Wallis test, all others with one-way analyses of variance.

**Table S7. Results of Regression Models Predicting Negative Symptom Severity from Win-Stay Parameter with Clinical and Demographic Covariates.**

| **Model 1.** |  |  |  |  |
| --- | --- | --- | --- | --- |
| **Omnibus** | **R^2^** | **F (df)** | **p** |  |
|  | .195 | 17.58 (6, 435) | < .001 |  |
| **Predictor** | **B (sb)** | **t** | **p** |  |
| Win-stay_G_ | -0.09 (.04) | -2.34 | **.020** |  |
| CHR status | -0.86 (1.01) | -0.85 | .397 |  |
| Positive symptoms | 0.14 (0.11) | 1.27 | .204 |  |
| Depressive symptoms | 0.37 (0.04) | 8.32 | **< .001** |  |
| Age | 0.00 (0.09) | .01 | .993 |  |
| Sex | -2.45 (0.74) | -3.32 | **< .001** |  |
|  |  |  |  |  |
| **Model 2.** |  |  |  |  |
| **Omnibus** | **R^2^** | **F (df)** | **p** |  |
|  | .212 | 11.56 (10, 431) | < .001 |  |
| **Predictor** | **B (sb)** | **t** | **p** |  |
| Win-stay_G_ | -0.08 (0.04) | -1.98 | **.048** |  |
| CHR status | -0.85 (1.0) | -0.85 | .399 |  |
| Positive symptoms | 0.14 (0.11) | 1.28 | .203 |  |
| Depressive symptoms | 0.37 (0.04) | 8.37 | **< .001** |  |
| Age | -0.01 (0.09) | -0.16 | .871 |  |
| Sex | -2.35 (0.74) | -3.19 | **.002** |  |
| Win-stay_G_ × age | -0.00 (0.01) | -0.40 | .688 |  |
| Win-stay_G_ × sex | -0.08 (0.08) | -1.06 | .288 |  |
| Age × sex | 0.10 (0.18) | 0.56 | .579 |  |
| Win-stay_G_ × age × sex | 0.05 (0.02) | 2.55 | **.011** |  |
|  |  |  |  |  |
| **Simple Effects** |  |  |  |  |
|  |  |  |  |  |
| **Effect of Win-stay_G_ × age interaction among males vs. females** | | | | |
| Male | -0.04 | 2.75 | **.006** |  |
| Female | 0.01 | 0.90 | .369 |  |
| **Age differences in effect of win-stay_G_ among males vs. females** | | | | |
| **Male** |  |  |  |  |
| Younger | 0.13 (0.09) | 1.43 | .154 |  |
| Mean | -0.02 (0.06) | -0.32 | .748 |  |
| Older | -0.17 (0.07) | -2.28 | **.023** |  |
| **Female** |  |  |  |  |
| Younger | -0.15 (0.07) | -2.26 | **.024** |  |
| Mean | -0.10 (0.05) | -2.15 | **.032** |  |
| Older | -0.04 (0.08) | -0.68 | .497 |  |

*Note*. Regression terms for Model 2 were centered to aid interpretability. For simple effects of win-stay_G_ at different ages for males vs. females, the mean age was 23.5. Younger age was centered at 19.5 (1 standard deviation below the mean) and older age was centered at 27.5 (1 standard deviation above the mean).

**Table S8. Results of Regression Models Predicting Depressive Symptom Severity from Win-Stay Parameter with Clinical and Demographic Covariates.**

| **Model 1.** |  |  |  |
| --- | --- | --- | --- |
| **Omnibus** | **R^2^** | **F (df)** | **p** |
|  | .212 | 19.52 (6, 435) | < .001 |
| **Predictor** | **B (sb)** | **t** | **p** |
| Win-stay_G_ | 0.04 (0.04) | 1.10 | .271 |
| CHR status | -0.70 (1.02) | -0.68 | .495 |
| Positive symptoms | 0.26 (0.11) | 2.38 | **.018** |
| Negative symptoms | 0.37 (0.05) | 8.32 | **< .001** |
| Age | 0.14 (0.09) | 1.55 | .122 |
| Sex | 2.34 (0.75) | 3.26 | .001 |
|  |  |  |  |
| **Model 2.** |  |  |  |
| **Omnibus** | **R^2^** | **F (df)** | **p** |
|  | .223 | 12.38 (10, 431) | < .001 |
| **Predictor** | **B (sb)** | **t** | **p** |
| Win-stay_G_ | 0.04 (0.04) | 0.91 | .37 |
| CHR status | -0.72 (1.02) | -0.71 | .478 |
| Positive symptoms | 0.24 (0.11) | 2.26 | **.023** |
| Negative symptoms | 0.38 (0.05) | 8.37 | **< .001** |
| Age | 0.12 (0.09) | 1.38 | .168 |
| Sex | 2.41 (0.75) | 3.23 | **.001** |
| Win-stay_G_ × age | 0.00 (0.011) | 0.07 | .943 |
| Win-stay_G_ × sex | -0.03 (0.08) | -0.39 | .695 |
| Age × sex | -0.36 (0.18) | -2.04 | **.042** |
| Win-stay_G_ × age × sex | -0.03 (0.02) | -1.30 | .194 |

*Note*. CHR status refers to clinical high-risk vs. clinical control.

**Table S9. Correlations between Primary Behavioral Parameters and Negative Symptom Factors.**

| **Parameter** | **NSI-PR Factors** | | | |
| --- | --- | --- | --- | --- |
|  | **Motivation and Pleasure** | | **Diminished Expression** | |
|  | **r/ρ** | **p** | **ρ** | **p** |
| **Win-stay_G_** | -.11 | **.018** | -.13 | **.002** |
| **Lose-shift_G_** | -.08 | .085 | -.07 | .113 |
| **Win-stay_LA_** | -.07 | .131 | -.05 | .289 |
| **Lose-shift_LA_** | -.09 | **.035** | -.04 | .441 |

*Note.* Correlations involving the win-stay_G_ parameter were determined with the Spearman method; all other analyses involving the Motivation and Pleasure factor used the Pearson method.

**Table S10. Results of Regression Models Predicting Negative Symptom Severity from Win-Stay Parameter and Neurocognitive Covariates.**

| **Model 1.** |  |  |  |
| --- | --- | --- | --- |
| **Omnibus** | **R^2^** | **F (df)** | **p** |
|  | .03 | 7.27 (2, 444) | < .001 |
| **Predictor** | **B (sb)** | **t** | **p** |
| Win-stay_G_ | -0.10 (0.03) | -3.13 | **.002** |
| WRAT | -0.04 (0.03) | -1.37 | .171 |
|  |  |  |  |
| **Model 2.** |  |  |  |
| **Omnibus** | **R^2^** | **F (df)** | **p** |
|  | .06 | 5.49 | < .001 |
| **Predictor** | **B (sb)** | **t** | **p** |
| Win-stay_G_ | -0.08 (0.03) | -2.39 | **.017** |
| WRAT | -0.01 (0.03) | -0.22 | .827 |
| BACS | -0.08 (.04) | -2.40 | **.017** |
| HVLT | -0.12 (0.10) | -1.25 | .212 |

**Table S11. Overall Results of Task Performance During the Learning Phase Alone and During the Learning and Transfer Phases**

| **Variable** | **Learning Phase Only** | | **Learning Phase & Transfer Phase** | |
| --- | --- | --- | --- | --- |
|  | **F (df)** | **p** | **F (df)** | **p** |
| **Group** | 0.81 (2, 705) | .447 | 0.57 (1, 705) | .565 |
| **Block** | 209.46 (2.59, 1832.5) | **< .001** | 234.78 (3.4, 2407.2) | **< .001** |
| **Probability** | 116.74 (1, 707) | **< .001** | 123.62 (1, 707) | **< .001** |
| **Valence** | 3.93 (1, 707) | **.048** | 4.59 (1, 707) | **.032** |
| **Block × probability** | 1.02 (2.71, 1912.7) | .378 | 0.75 (3.47, 2455.9) | .539 |
| **Block × valence** | 0.49 (2.79, 1972.5) | .674 | 0.35 (3.57, 2524) | .826 |
| **Group × block** | 1.04 (5.19, 1827.4) | .394 | 2.08 (6.8, 2399.8) | **.044** |
| **Group × probability** | 1.61 (2, 705) | .201 | 1.73 (2, 705) | .178 |
| **Group × valence** | 2.93 (2, 705) | .054 | 2.74 (2, 705) | .066 |
| **Block × probability × valence** | 0.47 (2.75, 1945.7) | .689 | 1.70 (3.65, 2583.5) | .153 |
| **Block × probability × group** | 0.25 (5.42, 1903.8) | .951 | 0.26 (6.94, 2447) | .968 |
| **Block × valence × group** | 0.76 (5.58, 1963) | .593 | 0.56 (7.14, 2520.8) | .791 |
| **Probability × valence × group** | 0.26 (2, 705) | .771 | 0.13 (2, 705) | .881 |
| **Block × probability × valence × group** | 0.45 (5.5, 1937.8) | .830 | 0.48 (7.3, 2573.7) | .873 |

*Note*. Degrees of freedom and p-values adjusted with the Huynh-Feldt correction when sphericity assumptions were violated.

Percent Optimal Choice

|  | CHR | CC | HC |
| --- | --- | --- | --- |
| Block 1 | 72.962 | 72.293 | 71.771 |
| Block 2 | 80.659 | 79.243 | 80.4 |
| Block 3 | 83.245 | 80.778 | 81.386 |
| Block 4 | 84.015 | 82.469 | 84.129 |
| Test | 86.614 | 86.857 | 89.165 |

**Figure S1. Accuracy rate across blocks of the Learning/Acquisition phase and in the Transfer/Test phase.**

*Note*: Transfer/Test results reflect choices made for familiar stimulus pairings but without feedback. Error bars represent standard deviation.

**Table S12. Means, Standard Deviations, and Results of Between- and Within-Group Analyses for Transfer Phase Measures of the Reinforcement Learning Task.**

| **Parameter** | **CHR** | **CC** | **HC** |  |  |  |
| --- | --- | --- | --- | --- | --- | --- |
|  | **Mean (SD)** | | | **Test Statistic (df)** | **p** |  |
| **FW-FL** | 85.76 (18.53) | 86.93  (17.8) | 87.07 (19.22) | 0.38 (2, 705) | .682 |  |
| **FW-FLA** | 54.54 (25.19) | 56.17 (25.89) | 53.17 (26.82) | 0.71 (2, 705) | .491 |  |
| **IW-FLA** | 84.16 (20.13) | 83.64  (20.2) | 85.5  (20.52) | 0.44 (2, 705) | .644 |  |
|  | **NSI-PR Total and Factors** | | | | | |
|  | **NSI-PR total** | | **Motivation and Pleasure** | | **Diminished Expression** | |
|  | **r** | **p** | **r** | **p** | **ρ** | **p** |
| **FW-FL** | -.12 | **.009** | -.13 | **.003** | -.03 | .489 |
| **FW-FLA** | .028 | .532 | .00 | .931 | -.01 | .845 |
| **IW-FLA** | -.09 | **.045** | -.09 | **.049** | .03 | .572 |

*Note*. Correlations between task parameters and negative symptoms were conducted in the combined clinical sample.

**Table S13. Correlations Among Computational and Behavioral Parameters in the Combined Healthy and Clinical Sample.**

|  | **2** | **3** | **4** | **5** | **6** | **7** |
| --- | --- | --- | --- | --- | --- | --- |
| **1. α_P_** | .118** | -.349*** | .185*** | .151*** | .156*** | .069^a^ |
| **2. α_N_** | 1 | -.151*** | -.108** | -.017 | .196*** | .128** |
| **3. β** |  | 1 | .538*** | .096* | .333*** | .183*** |
| **4. Win-stay_G_** |  |  | 1 | - | - | - |
| **5. Lose-shift_G_** |  |  |  | 1 | - | - |
| **6. Win-stay_LA_** |  |  |  |  | 1 | - |
| **7. Lose-shift_LA_** |  |  |  |  |  | 1 |

*Note.* β binarized based on median split due to extreme skewness and kurtosis. Correlations among behavioral parameters (e.g., win-stay_G_, lose-shift_G_) reported in Table 2 of the main text.

^a^*p* < .10, **p* < .05, ***p* < .01, ****p* < .001.

**Table S14. Results of Regression Models Predicting Behavioral Parameters from Computational Parameters in the Combined Healthy and Clinical Sample.**

| **Model 1.** |  |  |  |
| --- | --- | --- | --- |
| **DV: Win-Stay_G_** |  |  |  |
| **Omnibus** | **R^2^** | **F (df)** | **p** |
|  | .282 | 64.59 (4,657) | < .001 |
| **Predictor** | **B (sb)** | **t** | **p** |
| α_P_ | 10.01 (0.97) | 10.31 | **< .001** |
| α_N_ | -2.13 (1.04) | -2.05 | **.040** |
| β | 9.89 (0.67) | 14.67 | **< .001** |
| Clinical status | -0.36 (0.72) | -0.50 | .617 |
|  |  |  |  |
| **Model 2.** |  |  |  |
| **DV: Lose-Shift_G_** |  |  |  |
| **Omnibus** | **R^2^** | **F (df)** | **p** |
|  | .051 | 8.65 (4, 648) | < .001 |
| **Predictor** | **B (sb)** | **t** | **p** |
| α_P_ | 13.45 (2.55) | 5.27 | **< .001** |
| α_N_ | -1.54 (2.69) | -0.57 | .567 |
| β | 7.40 (1.87) | 4.21 | **< .001** |
| Clinical status | 1.23 (1.87) | 0.66 | .512 |
|  |  |  |  |
| **Model 3.** |  |  |  |
| **DV: Win-Stay_LA_** |  |  |  |
| **Omnibus** | **R^2^** | **F (df)** | **p** |
|  | .250 | 54.38 (4, 652) | < .001 |
| **Predictor** | **B (sb)** | **t** | **p** |
| α_P_ | 8.77 (1.08) | 8.09 | **< .001** |
| α_N_ | 7.78 (1.15) | 6.76 | **< .001** |
| β | 9.67 (0.75) | 12.86 | **< .001** |
| Clinical status | -1.03 (0.81) | -1.28 | .201 |
|  |  |  |  |
| **Model 4.** |  |  |  |
| **DV: Lose-Shift_LA_** |  |  |  |
| **Omnibus** | **R^2^** | **F (df)** | **p** |
|  | .078 | 13.70 (3, 482) | < .001 |
| **Predictor** | **B (sb)** | **t** | **p** |
| α_P_ | 8.25 (2.37) | 3.48 | **< .001** |
| α_N_ | 9.62 (2.52) | 3.82 | **< .001** |
| β | 10.11 (1.64) | 6.17 | **< .001** |
| Clinical status | -2.74 (1.76) | -1.56 | .119 |

*Note*. Clinical status refers to healthy control versus combined clinical group. β binarized based on median split due to extreme skewness and kurtosis.

**Table S15. Results of Regression Models Predicting Win-Stay_G_ Parameter from Computational Parameters and their Interaction in the Combined Healthy and Clinical Sample.**

| **Omnibus** | **R^2^** | **F (df)** | **p** |
| --- | --- | --- | --- |
|  | .290 | 53.47 (5, 656) | < .001 |
| **Predictor** | **B (sb)** | **t** | **P** |
| α_P_ | 9.70 (0.97) | 9.96 | **< .001** |
| α_N_ | -1.98 (1.03) | -1.91 | .056 |
| β | 9.83 (0.67) | 14.63 | **< .001** |
| α_P_ × β | 5.04 (1.95) | 2.59 | **.010** |
| Clinical status | -0.38 (0.72) | -0.53 | .596 |
|  |  |  |  |
| **Simple Effects** |  |  |  |
| Low β | 7.18 (1.46) | 4.92 | **< .001** |
| High β | 12.22 (1.29) | 9.48 | **< .001** |

*Note*. Clinical status refers to healthy control versus combined clinical group. β binarized based on median split due to extreme skewness and kurtosis.

**Table S16. Results of Regression Analyses Predicting Transfer-Phase Preference for Frequent Winners vs. Frequent Loss-Avoiders from Computational Parameters.**

| **Stratified Models** |  |  |  |
| --- | --- | --- | --- |
|  |  |  |  |
| **Model 1.** |  |  |  |
| **Group: HC** |  |  |  |
| **Omnibus** | **R^2^** | **F (df)** | **p** |
|  | .035 | 1.99 | .117 |
| **Predictor** | **B (sb)** | **t** | **p** |
| α_P_ | 4.83 (6.48) | 0.75 | .457 |
| α_N_ | -5.83 (6.91) | -0.84 | .400 |
| β | 9.62 (4.49) | 2.14 | **.034** |
|  |  |  |  |
| **Model 2.** |  |  |  |
| **Group: Clinical** |  |  |  |
| **Omnibus** | **R^2^** | **F (df)** | **p** |
|  | .022 | 3.76 | .011 |
| **Predictor** | **B (sb)** | **t** | **p** |
| α_P_ | -8.35 (3.55) | -2.36 | **.019** |
| α_N_ | -7.35 (3.79) | -1.94 | .053 |
| β | -4.65 (2.45) | -1.89 | .059 |
|  |  |  |  |
| **Combined Model** |  |  |  |
|  |  |  |  |
| **Model 3.** |  |  |  |
| **Group: Combined** |  |  |  |
| **Omnibus** | **R^2^** | **F (df)** | **p** |
|  | .023 | 3.08 (5, 656) | .009 |
| **Predictor** | **B (sb)** | **t** | **p** |
| Clinical status | 8.35 (3.33) | 2.51 | **.012** |
| α_P_ | -5.14 (3.11) | -1.66 | .098 |
| α_N_ | -7.48 (3.32) | -2.26 | **.024** |
| β | 6.96 (4.01) | 1.71 | .089 |
| Clinical status × β | -10.86 (4.62) | -2.35 | **.019** |
|  |  |  |  |
| **Simple Effects** |  |  |  |
| HC | 6.96 (4.08) | 1.71 | .089 |
| Clinical | -3.90 (2.45) | -1.59 | .112 |

*Note*. Clinical status refers to healthy control versus combined clinical group. β binarized based on median split due to extreme skewness and kurtosis.

**Table S17. Means, Standard Deviations, and Results of Between-Group Analyses and Within-Group Symptom Correlations for Computational Parameters.**

| **Parameter** | **CHR** | **CC** | **HC** |  |  |
| --- | --- | --- | --- | --- | --- |
|  | **Mean (SD)** | | | **Test Statistic (df)** | **p** |
| **α_P_** | .56 (.35) | .56 (.34) | .54 (.35) | 0.23 (2, 653) | .794 |
| **α_N_** | .39 (.31) | .44 (.31) | .41 (.31) | 1.30 (2, 653) | .274 |
| **β** | .09 (.21) | .07 (.16) | .10 (.22) | 0.87 (2) | .646 |
|  | **Correlations Between Computational Parameters and Symptom Severity** | | | |  |
|  | **Negative Symptoms** | | **Depressive Symptoms** | |  |
|  | **r** | **p** | **r** | **p** |  |
| **α_P_** | -.06 | .203 | -.04 | .359 |  |
| **α_N_** | .00 | .949 | .02 | .747 |  |
| **β** | -.01 | .868 | .00 | .968 |  |

*Note*. Statistical comparisons of the three study groups on α parameters were conducted with analyses of variance. Means and standard deviations are provided for the β parameter for descriptive purposes, however the statistical test of between-group differences was conducted on the median-split transformation using a chi-square analysis due to extreme skewness and kurtosis of the untransformed β. Correlations between task parameters and negative symptoms were conducted in the combined clinical sample.

**Table S18. Results of Regression Models Testing Age and Sex-Dependent Associations Between α_P_ and Negative Symptom Severity.**

| **Omnibus** | **R^2^** | **F (df)** | **p** |  |
| --- | --- | --- | --- | --- |
|  | .198 | 10.66 (10, 431) | < .001 |  |
| **Predictor** | **B (sb)** | **t** | **p** |  |
| α_P_ | -0.99 (1.02) | -.98 | .327 |  |
| CHR status | -0.80 (1.02) | -0.78 | .434 |  |
| Positive symptoms | 0.14 (1.01) | 1.29 | .198 |  |
| Depressive symptoms | 0.36 (0.04) | 8.17 | **< .001** |  |
| Age | 0.01 (0.09) | 0.14 | .892 |  |
| Sex | -2.41 (0.74) | -3.25 | **.001** |  |
| α_P_ × age | 0.27 (0.26) | 1.06 | .291 |  |
| α_P_ × sex | -0.13 (2.07) | -0.06 | .949 |  |
| Age × sex | 0.02 (0.18) | 0.09 | .929 |  |
| α_P_ × age × sex | -0.96 (0.50) | -1.92 | .055 |  |
|  |  |  |  |  |
| **Simple Effects** |  |  |  |  |
|  |  |  |  |  |
| **Effect of α_P_ × age interaction among males vs. females** | | | | |
| Male | 0.90 | F =6.26 | **.013** |  |
| Female | -0.05 | F =0.02 | .883 |  |
|  |  |  |  |  |
| **Age differences in effect of α_P_ among males vs. females** | | | | |
| **Male** |  |  |  |  |
| Younger | -4.57 (2.27) | -2.02 | **.045** |  |
| Mean | -0.91 (1.63) | -0.56 | .579 |  |
| Older | 2.75 (2.11) | 1.30 | .194 |  |
| **Female** |  |  |  |  |
| Younger | -0.84 (1.90) | -0.44 | .660 |  |
| Mean | -1.04 (1.28) | -0.81 | .418 |  |
| Older | -1.24 (1.87) | -0.67 | .506 |  |

*Note*. CHR status refers to clinical high-risk vs. clinical control. For simple effects of α_P_ at different ages for males vs. females, the mean age was 23.5. Younger and older ages were centered at 1 standard deviation below and above the mean, respectively.

**Table S19. Results of Regression Models Testing Age and Sex-Dependent Associations Between α_N_ and Negative Symptom Severity.**

| **Omnibus** | **R^2^** | **F (df)** | **p** |
| --- | --- | --- | --- |
|  | .207 | 11.22 (10, 431) | < .001 |
| **Predictor** | **B (sb)** | **t** | **p** |
| α_N_ | -0.05 (1.14) | -0.04 | .967 |
| CHR status | -0.54 (1.01) | -0.54 | .588 |
| Positive symptoms | 0.17 (0.11) | 1.56 | .120 |
| Depressive symptoms | 0.37 (0.04) | 8.26 | **< .001** |
| Age | -0.01 (.09) | -0.13 | .895 |
| Sex | -2.45 (0.74) | -3.32 | **.001** |
| α_N_ × age | -0.03 (0.28) | -0.13 | .900 |
| α_N_ × sex | -0.11 (2.3) | -0.05 | .963 |
| Age × sex | 0.04 (0.18) | 0.22 | .828 |
| α_N_ × age × sex | -1.82 (0.54) | -3.35 | **.001** |
|  |  |  |  |
| **Simple Effects** |  |  |  |
|  |  |  |  |
| **Effect of of α_N_ × age on interaction among males vs. females** | | | |
| Male | 1.17 | F=8.46 | **.004** |
| Female | -0.65 | F=3.16 | .076 |
|  |  |  |  |
| **Age differences in effect of α_N_ among males vs. females** | | | |
| **Male** |  |  |  |
| Younger | -4.69 (2.61) | -1.80 | .073 |
| Mean | 0.03 (1.40) | 0.01 | .989 |
| Older | 4.75 (2.44) | 1.95 | .052 |
| **Female** |  |  |  |
| Younger | 0.34 (2.53) | 1.29 | .199 |
| Mean | -0.08 (1.40) | -0.06 | .953 |
| Older | -2.70 (2.10) | -1.29 | .199 |

*Note*. CHR status refers to clinical high-risk or clinical control. For simple effects of α_N_ at different ages for males vs. females, the mean age was 23.5. Younger and older ages were centered at 1 standard deviation below and above the mean, respectively.

**Table S20. Results of Regression Models Testing Age and Sex-Dependent Associations Between β and Negative Symptom Severity.**

| **Omnibus** | **R^2^** | **F (df)** | **p** |
| --- | --- | --- | --- |
|  |  |  |  |
| **Predictor** | **B (sb)** | **t** | **p** |
| β | -0.04 (0.67) | -0.06 | .952 |
| CHR status | -0.67 (1.01) | -0.66 | .508 |
| Positive symptoms | 0.15 (0.11) | 1.41 | .16 |
| Depressive symptoms | 0.36 (0.04) | 8.13 | **< .001** |
| Age | -0.01 (0.09) | -0.06 | .905 |
| Sex | -2.36 (0.74) | -3.20 | **.002** |
| β × age | -0.11 (0.17) | -0.65 | .514 |
| β × sex | -0.81 (1.47) | -0.55 | .581 |
| Age × sex | 0.09 (0.18) | 0.51 | .614 |
| β × age × sex | 1.35 (0.35) | 3.85 | **< .001** |
|  |  |  |  |
| **Simple Effects** |  |  |  |
|  |  |  |  |
| **Effect of β × age interaction among males vs. females** | | | |
| Male | -1.01 | F=13.97 | **< .001** |
| Female | 0.34 | F=2.32 | .129 |
|  |  |  |  |
| **Age differences in effect of β among males vs. females** | | | |
| **Male** |  |  |  |
| Younger | 4.56 (1.66) | 2.74 | **.006** |
| Mean | 0.49 (1.19) | 0.41 | .678 |
| Older | -3.57 (1.56) | -2.28 | **.023** |
| **Female** |  |  |  |
| Younger | -1.69 (1.21) | -1.39 | .165 |
| Mean | -0.31 (0.84) | -0.37 | .711 |
| Older | 1.06 (1.26) | 0.84 | .403 |

*Note*. CHR status refers to clinical high-risk or clinical control. For simple effects of β at different ages for males vs. females, the mean age was 23.5. Younger and older ages were centered at 1 standard deviation below and above the mean, respectively.
